# Submillimeter diffusion MRI using an in-plane segmented 3D multi-slab acquisition and denoiser-regularized reconstruction

**DOI:** 10.1016/j.media.2025.103834

**Published:** 2026-01

**Authors:** Ziyu Li, Silei Zhu, Karla L. Miller, Wenchuan Wu

**Affiliations:** Oxford Centre for Integrative Neuroimaging, FMRIB, Nuffield Department of Clinical Neurosciences, University of Oxford, Oxford, United Kingdom

**Keywords:** 3D diffusion MRI, High-resolution, Tractography, Gyral bias, U-fiber, White matter

## Abstract

Diffusion MRI (dMRI) enables brain connectivity mapping but is constrained by spatial resolution. Previous post-mortem studies have demonstrated the potential of submillimeter dMRI in enabling more precise delineations of curved and crossing white matter pathways. However, achieving such resolution in-vivo poses significant challenges due to the intrinsically low signal-to-noise ratio (SNR). Furthermore, for echo-planar imaging (EPI), large matrix sizes often require long echo spacing, readout duration, and echo times (TE), leading to significant image distortion, T2* blurring, and T2 signal decay. Here, we propose an acquisition and reconstruction framework to overcome these challenges. Based on numerical simulations, we employ in-plane segmented 3D multi-slab EPI that leverages the optimal SNR efficiency of 3D multi-slab imaging while reducing echo spacing, readout durations, and TE using in-plane segmentation. This approach minimizes distortion, improves image sharpness, and enhances SNR. Additionally, we develop a denoiser-regularized reconstruction to suppress noise while maintaining data fidelity, which reconstructs high-SNR images without introducing substantial blurring or bias. At 3T, we present 0.53–0.65 mm in-vivo data that reveal finer fiber architectures, reduced gyral bias, and improved U-fiber mapping compared to 1.22 mm data. At 7T, we acquire 0.61 mm data that show excellent agreement with high-resolution post-mortem dMRI, demonstrating robustness and high SNR at an ultra-high field. Our method is implemented using the open-source, scanner-agnostic framework Pulseq to facilitate broader adoption across scanner platforms to benefit a wider range of applications. These results establish our approach as a promising tool for high-resolution dMRI, advancing neuroanatomical investigations of white matter architecture.

## 1 Introduction

High-resolution diffusion MRI (dMRI) offers a powerful tool for detailed investigation of white matter connectivity. The benefits of submillimeter dMRI have been demonstrated in post-mortem studies (Foxley et al., 2014; Miller et al., 2011; Roebroeck et al., 2019), where it provides more precise delineations of curved and crossing white matter pathways (e.g., transverse pontine fibers) compared to conventional resolutions (e.g., 2 mm). Additionally, submillimeter dMRI may help address a known challenge in dMRI fiber tracking (tractography) called “gyral bias”, where tracked fibers tend to terminate at gyral crowns instead of accurately capturing the sharp turn to the gyral walls (Fritz et al., 2019; Reveley et al., 2015; Schilling et al., 2018; Sotiropoulos and Zalesky, 2019; Van Essen et al., 2014). Higher spatial resolutions are also advantageous for the identification of short cortical association fibers (Song et al., 2014), commonly known as U-fibers, which connect cortical regions between adjacent gyri (Schmahmann and Pandya, 2009). U-fibers are particularly important for studying brain development, function, and pathology (d’Albis et al., 2018; Van Dyken et al., 2024). Furthermore, high-resolution dMRI holds great promise for precisely delineating small but crucial subcortical structures, as evidenced by post-mortem studies (Roebroeck et al., 2019). This includes detailed depiction of intersecting fibers in regions like the optic chiasm (Wedeen et al., 2008), cerebellar folia (Dell’Acqua et al., 2013; Wedeen et al., 2008) and brainstem (Aggarwal et al., 2013; Tendler et al., 2022; Wedeen et al., 2008), as well as precise localization of electrode targets in deep brain stimulation (Calabrese et al., 2015).

However, to harness these benefits of high-resolution dMRI in vivo requires overcoming considerable challenges. The image-forming mechanisms of conventional 2D single-shot echo-planar imaging (ss-EPI), the standard dMRI acquisition method in research and clinical practice, are fundamentally at odds with the requirements of high spatial resolution (Wu and Miller, 2017). A major hurdle is the intrinsically limited SNR associated with small voxel sizes. In 2D ss-EPI, this SNR limitation is exacerbated by the need for longer echo times (TE) to acquire more phase-encode lines, reducing signal strength, and longer repetition times (TR) to acquire more slices, lowering scan efficiency. Additionally, the protocol changes necessary to encode higher-resolution images incur compromises to voxel definition, counteracting the goals of high-resolution imaging. First, in the readout direction, increasing resolution requires longer echo spacing, leading to increased image distortion and T2* blurring. Second, excitation of thin slices is limited by maximum gradient strength, potentially leading to compromised slice profiles (Bernstein et al., 2004).

Advanced acquisition strategies have been proposed to mitigate these challenges in 2D ss-EPI. In-plane acceleration can shorten the echo time (reducing signal decay), readout duration (reducing blurring), and effective echo spacing (reducing distortion), but at the cost of increased noise from under-sampled reconstruction (Griswold et al., 2002; Pruessmann et al., 1999). Simultaneous multi-slice imaging (SMS) (Feinberg and Setsompop, 2013; Moeller et al., 2010; Setsompop et al., 2012) has been proposed to reduce TR and improve SNR efficiency using multiband excitations, though with the trade-off of higher noise level and increased RF power deposition. Combining in-plane acceleration and SMS further enhances scan efficiency, but the achievable acceleration factors are limited by intrinsic SNR loss and g-factor penalties. Recently, studies have proposed super-resolution-based approaches that have shown potential in enabling submillimeter dMRI. gSlider uses 2D acquisitions to excite a thin slab multiple times with distinct RF pulses, allowing a few (~5) thin slices to be resolved from the slab (Jun et al., 2024; Liao et al., 2021, 2023; Setsompop et al., 2018). Coupled with the SMS, gSlider-SMS can effectively shorten the TR and improve SNR efficiency. More recent work proposed the acquisition of multiple thick-slice volumes with rotated field-of-view (FOV) and super-resolution reconstruction to achieve submillimeter dMRI (Dong et al., 2024). However, an important consideration in super-resolution-based methods is the potential blurring effect that can result from the use of conditioning techniques (e.g., Tikhonov regularization (Dong et al., 2024; Setsompop et al., 2018)) when solving the inverse problem. More generally, despite achieving shorter TRs than conventional 2D ss-EPI, these methods cannot achieve the optimal TR range for SNR efficiency (TR=1–2 s).

High-resolution 3D dMRI acquisitions offer another effective pathway to shorten TR and resolve thin slices. By adding gradient encoding along the slice-selection direction, 3D methods introduce an orthogonal Fourier basis, improving both SNR and voxel shape fidelity compared to super-resolution methods. Typically, 3D acquisitions use multi-shot EPI readouts to efficiently cover 3D k-space (Miller et al., 2011). However, for in-vivo 3D single-slab acquisitions, accurately correcting for spatially varying, motion-induced phase errors for each 3D shot remains challenging. Recent studies have addressed this issue using self-navigated radial readouts (Feizollah and Tardif, 2025) or low-resolution 3D navigators (Maeng et al., 2025). Nevertheless, pure 3D approaches often require shorter-than-optimal TRs (<1 s (Maeng et al., 2025)), reducing SNR efficiency. Additionally, non-Cartesian trajectories employed to address motion sensitivity (Feizollah and Tardif, 2025) may be susceptible to blurring induced by magnetic field inhomogeneities, often necessitating additional correction strategies (Feizollah and Tardif, 2023).

An alternative acquisition approach for achieving high-resolution, SNR-efficient dMRI is 3D multi-slab imaging. Unlike pure 2D or 3D methods described above, 3D multi-slab imaging divides the whole brain into several slabs, each with a thickness of less than 2 cm to ensure that motion-induced phase variations can be effectively captured by a 2D navigator (Engström and Skare, 2013; Frank et al., 2010). Within each slab, 3D Fourier encoding is typically employed, offering high SNR and image sharpness. This hybrid approach is compatible with optimal TRs of 1–2 s (Engström and Skare, 2013; Frost et al., 2014; Wu, Poser, et al., 2016). However, conventional 3D multi-slab imaging often uses a single-shot EPI readout to cover one kz plane, resulting in extended TE and readout durations, which can lead to significant SNR penalties and T2* blurring, particularly at submillimeter resolutions.

Recent advances in image reconstruction have also played a critical role in enabling high-resolution dMRI, particularly through the use of SNR-enhancing regularizations. For example, in gSlider reconstruction, regularizations promoting spatial smoothness (Haldar et al., 2020) and transform-domain sparsity (e.g., spherical ridgelets (Ramos-Llorden et al., 2020)) have been introduced to address the ill-posed inverse problem, thereby improving SNR in submillimeter dMRI. Deep learning-based denoising have also been integrated into diffusion MRI reconstruction, typically through model-based unrolled neural networks (NNs), which alternate between physics-informed forward encoding and NN-based image denoising steps. This strategy effectively boosts SNR while ensuring data consistency (Aggarwal et al., 2018). The SNR and image quality improvements provided by denoising-based regularizations have been comprehensively demonstrated in 2D multi-shot dMRI (Aggarwal et al., 2019; Cho et al., 2023; Hu et al., 2021). However, the use of advanced denoising regularizations remains relatively underexplored in the context of 3D dMRI. This gap has limited the full realization of the inherent SNR efficiency benefits offered by 3D multi-slab imaging for achieving high-quality submillimeter dMRI.

In this study, we present an acquisition and reconstruction frame-work designed to address these limitations and achieve submillimeter in-vivo dMRI with robust quality. We leverage 3D multi-slab imaging for superior SNR efficiency, with in-plane segmented EPI used to shorten echo spacing, readout durations, and TE, thereby reducing distortion, T2* blurring, and improving SNR. A novel denoiser-regularized reconstruction is proposed for the segmented 3D multi-slab dMRI to effectively suppress noise during reconstruction while maintaining consistency with the acquired data to minimize reconstruction-induced blurring and biases. We present in-vivo experiments at 3T and 7T, producing high-quality submillimeter dMRI with protocols ranging from 0.53–0.65 mm isotropic resolutions. Tractography of the acquired submillimeter datasets compared to 1.22 mm resolution data reveal significantly finer fiber architectures, underscoring our method’s potential to address the gyral bias and improve U-fiber tracking. These findings hold considerable promise for advancing neuroanatomical investigation into the human brain.

## 2 Methods

### 2.1 In-plane segmented 3D multi-slab acquisition

Here, we use simulations to assess the impact of protocol decisions on high-resolution dMRI using 3D multi-slab acquisitions. In conventional 3D multi-slab diffusion imaging, each shot typically covers an entire kz plane using a single-shot EPI readout for efficient data acquisition (Engström and Skare, 2013; Wu, Poser, et al., 2016). However, for high-resolution EPI with a large matrix size, this single-shot acquisition can result in extended echo spacing, readout durations, and TE, leading to significant image distortion, T2* blurring, and reduced SNR. In-plane segmented multi-shot acquisitions hold promise in mitigating these issues. The most common approach is phase-encoding segmented EPI, where each shot undergoes a high under-sampling factor along the phase encoding direction, ky, (i.e., acquiring a regularly spaced subset of lines in each segment). This effectively shortens the effective echo spacing, readout duration, and TE. With phase-encoding segmentation, the effective echo spacing and associated image distortion decreases inversely proportional to the number of in-plane segments (*N_seg_*).

We performed simulations to quantitatively assess how effective resolution and SNR are affected by *N_seg_* at 0.6 mm and 1 mm resolutions at 3T and 7T, assuming unaccelerated acquisitions (i.e., with effective acceleration factor *R_eff_* = *N_seg_*/*N_acq_* = 1, where *N_acq_* is the number of acquired segments). We extended an existing single-shot simulation framework (Feizollah and Tardif, 2023) for multi-segment acquisitions, modifying the parameters to reflect realistic 3D multi-slab dMRI acquisitions: FOV=220×220×120 mm^3^, TR=2.5 s, maximum gradient amplitude *G_max_*=80 mT/m, RF excitation duration=6 ms, RF refocusing duration=10 ms, b-value=1000 s/mm^2^, and bandwidths of 992 Hz/pixel and 1384 Hz/pixel for 0.6 mm and 1 mm acquisitions, respectively. The T1, T2, and T2* values are set to 832/79.6/53.2 ms at 3T and 1220/47/26.8 ms at 7T for white matter (Cox and Gowland, 2010; Peters et al., 2007; Rooney et al., 2007; Wansapura et al., 1999).

The effective resolution is quantified by the full-width-half-maximum (FWHM) of the point spread functions simulated with various acquisition parameters. For the partial Fourier (PF) sampling (PF=6/8), conjugate symmetric filling and zero-padding are investigated in image reconstruction. The SNR is quantified based on:

(1) $$SNR\propto {\left(B0\right)}^{1.65}\sqrt{N_{PE}N_{par}}\frac{\text{Δ}x\text{Δ}y\text{Δ}z}{\sqrt{BW}}e^{-\frac{TE}{T2}}\left(1-e^{-\frac{TR}{T1}}\right)$$

where B0 is the main magnetic field strength, and the supralinear dependence (~(B0)^1.65^) reflects empirical measurements of field-dependent SNR increases (Pohmann et al., 2016). Δ*x*, Δ*y*, and Δ*z* are the effective resolution along readout, phase-encoding, and slice-selection directions, respectively. The voxel volume is given by Δ*x*Δ*y*Δ*z*, with Δ*y* derived from the PSF FWHM, and Δ*x*, Δ*z* assumed to match the nominal resolution. BW is the receiver bandwidth per pixel. TE is simulated under different acquisition conditions. *N_PE_* and *N_par_* refer to the number of in-plane phase-encoding lines and the number of kz partitions per slab, both of which influence SNR by affecting acquisition time (Bernstein et al., 2004). Since the simulation assumes a matched FOV (220 × 220 × 120 mm^3^) and TR (2.5 s) for both 0.6 mm and 1 mm acquisitions, changes in *N_PE_* and *N_par_* scale proportionally with resolution. It is also worth noting that since this model assumes unaccelerated acquisitions (i.e., R_eff_ = 1), it does not explicitly model noise amplification from under-sampled reconstruction (i.e., g-factor). Additionally, the simulated real-valued PSFs are intended to characterize the blurring introduced by the k-space sampling alone. They do not fully capture the behavior of the actual, complex-valued MRI reconstruction which might lead to additional blurring or artifacts.

As detailed in the Results section, the simulations show that increasing *N_seg_* improves effective resolution and SNR. To achieve satisfactory effective resolution and SNR while keeping the scan duration per volume within a reasonable range to accommodate more diffusion directions, we select *N_seg_* values of 6 for 3T and 8 for 7T, ensuring a practical balance between image quality and acquisition efficiency.

For the sampling order of in-plane segmented 3D EPI, we choose to acquire all in-plane ky segments of a given kz in immediate succession before moving to the next kz (depicted as “ky-kz” in Supplementary Fig. 1a). Compared to the alternative order where all kz planes for one ky segment are acquired before proceeding to the next segment (“kz-ky” in Supplementary Fig. 1b), our chosen sampling order reduces the inconsistencies between in-plane segments, improving motion robustness and reducing image artifacts (Ivanov et al., 2015; Polimeni et al., 2016), especially for the *b =* 0 image acquisition due to the pronounced cerebrospinal fluid (CSF) signal aliasing introduced by inter-segment inconsistency (Supplementary Fig. 1).

### 2.2 In-vivo experiments

We adapted a 3D multi-slab spin-echo diffusion MRI sequence (Wu, Poser, et al., 2016) to incorporate in-plane segmented acquisitions following the sampling order illustrated in Supplementary Fig. 1a. The sequence was implemented on the Siemens platform as well as on an open-source, scanner agnostic framework “Pulseq” (Layton et al., 2017), with this latter implementation aiming to promote broader accessibility and applications. In-vivo experiments at 3T (Siemens Prisma, Erlangen, Germany) and 7T (Siemens Magnetom, Erlangen, Germany) were conducted, both with 32-channel receive coils. Written informed consent was obtained from subjects before scanning in accordance with local ethics. Submillimeter dMRI data at 0.65 mm, 0.61 mm, and 0.53 mm isotropic resolutions were acquired using the following protocols, with key parameters listed in Table 1, and additional parameters listed in Supplementary Table 1:

#### 3T 0.65 mm protocol

This protocol aimed to validate the efficacy of the proposed acquisition and reconstruction framework and to establish a robust high-resolution dataset, albeit with a long scan time per diffusion direction. Fully sampled data were acquired at 0.65 mm isotropic resolution without acceleration or PF, resulting in a relatively long scan time per volume (6 min). Consequently, the number of diffusion directions was limited to six (along with two *b =* 0 images) within a 48-minute session.

#### 3T 0.53 mm protocol

This protocol aimed to provide a more practical solution by incorporating PF and acceleration (*R_eff_* =2) to reduce TE and scan time per volume, thereby improving SNR and enabling the acquisition of more diffusion directions for advanced diffusion analyses beyond diffusion tensor imaging (DTI). This protocol yielded data with 20 diffusion directions and four *b* = 0 images in a 65-minute session.

#### 7T 0.61 mm protocol

This protocol aimed to demonstrate the robustness of the method at an ultra-high field strength. Fully sampled data were acquired at 0.61 mm isotropic resolution at 7T without acceleration or PF. Due to the shorter T2 and T2* at 7T, a higher *N_seg_* of 8 was necessary to avoid excessive TE and readout durations to realize the SNR benefit at 7T and achieve high effective resolution. This increased *N_seg_* extended the scan time per diffusion direction, and the six-direction dataset (with two *b* =0 images) was acquired in 61 min.

#### Reference data at conventional resolutions

For comparison, reference dMRI datasets at conventional resolutions were acquired in separate sessions from the same subjects at both 3T and 7T At 3T, a dMRI dataset at 1.22 mm isotropic resolution was obtained using conventional 3D multi-slab EPI with the following parameters: matrix size=180×180×91, *R* = 3, PF=6/8, TE1/TE2/TR=70/150/2700 ms, effective echo spacing=0.27 ms, number of slabs=10, slices per slab =10, kz oversampling=20 %, number of overlapped slices between slabs=1, *T_acq_* per volume=32.4 s, 24 diffusion encoding directions (*b* = 1000 s/mm^2^) and 4 *b* = 0 images (with 2 images acquired along the opposite phase encoding direction), total scan time=15.1 min. At 7T, another dMRI dataset at 1.05 mm isotropic resolution was acquired using a blip-reversed 3D multi-slab acquisition (Li et al., 2023) with matrix size=210×210×115, 48 diffusion encoding directions (*b* = 1000 s/mm^2^) and 6 *b* = 0 images, *R* = 3, TE1/TE2/TR=82/150/1800 ms, *T_acq_*=36 s per volume and ~33 min in total. Additionally, a T1-weighted (T1w) anatomical image at 0.7 mm isotropic resolution was acquired at 3T using magnetization-prepared rapid gradient-echo imaging (MPRAGE) (Mugler III and Brookeman, 1990) following the protocol from the Human Connectome Project (HCP) (Glasser et al., 2016).

### 2.3 Denoiser-regularized reconstruction

High-resolution dMRI reconstruction is limited by the inherently low SNR. Image denoising is a common strategy to enhance SNR, with various methods available both for general natural images (Buades et al., 2011; Dabov et al., 2007; Maggioni et al., 2012) and specifically for dMRI (Moeller et al., 2021; Veraart et al., 2016). However, these methods typically function as standalone post-processing steps after image reconstruction, which can introduce biases and blurring during dMRI denoising (Manzano Patron et al., 2024).

Our approach integrates denoising within a SPIRiT-based (Lustig and Pauly, 2010) multi-shot reconstruction framework, aiming to suppress noise while minimizing biases and blurring by enforcing data consistency:

(2) $$\text{arg}\underset{x}{min}\sum_{i}^{N_{shot}}\|D_{i}FP_{i}F^{-1}x-y_{i}{\|}_{2}^{2}+\lambda_{1}\|(G-I)x{\|}_{2}^{2}+\lambda_{2}\|F^{-1}x-\text{Φ}\left(F^{-1}x\right){\|}_{2}^{2},$$

where *x* is the desired fully-sampled multi-coil k-space data to be reconstructed, *N_shot_* is the total number of shots, *D_i_* is the shot-sampling mask for the *i*^*th*^ shot, *F* is the Fourier Transform, *P_i_* is the motion-induced phase variance for the *i*^*th*^ shot, *y_i_* is the acquired data for the *i*^*th*^ shot, *G* is the SPIRiT kernel trained on coil calibration data, *I* is the identity matrix, Φ is the denoiser, and *λ*_1_, *λ*_2_ are the weights for SPIRiT and denoiser regularizations, respectively.

To efficiently solve Eq. (2), we leverage the “plug-and-play” approach (Ahmad et al., 2020), iteratively alternating image denoising with forward-model-based reconstruction:

(3) $$\begin{array}{l} x^{k}=\text{arg}\underset{x}{min}\sum_{i}^{N_{shot\;}}\|D_{i}FP_{i}^{H}F^{-1}x-y_{i}{\|}_{2}^{2}+\lambda_{1}\|(G-I)x{\|}_{2}^{2}\\ \;\;\;\;\;\;\;\;\;\;\;+\lambda_{2}\|F^{-1}x-z^{k-1}{\|}_{2}^{2},\\ z^{k}=\text{Φ}\left(F^{-1}x^{k}\right), \end{array}$$

which enables the incorporation of advanced denoisers, Φ (*F*^–1^ *x*^*k*^), while enforcing the data consistency by minimizing $\sum_{i}^{N_{shot\;}}\|D_{i}FP_{i}^{H}F^{-1}x-y_{i}{\|}_{2}^{2}$ and the consistency with the calibration data by minimizing the SPIRiT constraint $\|(G-I)x{\|}_{2}^{2}.$ The proposed denoiser-regularized SPIRiT reconstruction is referred to as “DnSPIRiT” hereafter.

The reconstruction was conducted in MATLAB 2021a (Mathworks, Natick, MA, USA). The SPIRiT kernel *G* in Eq. (3) was trained using a whole-brain gradient echo coil calibration scan (~2 min acquisition time). All 32-coil k-space data were compressed to 8 coils (Zhang et al., 2013).

The 2D navigator images for estimating shot-to-shot motion-induced phase variations were reconstructed with 2D GRAPPA (Griswold et al., 2002). Since the phase variations are expected to be spatially smooth, images were subsequently filtered using a k-space Hamming window of size 32×32 to reduce noise (Wu, Poser, et al., 2016). Phase images were then extracted as estimates of motion-induced phase errors. The reconstruction in Eq. (3) was performed for each ky-kz plane after the k-space data being first Fourier transformed along kx. The reconstructed 2D images were concatenated along the readout direction to form the whole image volume. The SPIRiT kernel size was set to 5 × 5. Eq. (3) was solved using a conjugate gradient (CG) method. The first iteration of Eq. (3) performed a standard SPIRiT reconstruction with motion-induced phase error correction.

For denoising in DnSPIRiT, the choice of method depended on the dataset. The most commonly used approach for dMRI denoising is PCA-based methods (Moeller et al., 2021; Veraart et al., 2016), which was applied to reconstruct the 20-direction dataset. However, for the 6-direction datasets, where redundancy across diffusion directions is insufficient for PCA-based methods, the image-space denoising method BM4D (Maggioni et al., 2012) was used instead.

For the 6-direction 3T 0.65 mm and 7T 0.61 mm protocols, BM4D was applied for 5 iterations (i.e., *N_iter_*=5) with hyperparameters empirically selected as *λ*_1_=10 and *λ*_2_=2. During each iteration, the sum-of-squares of the reconstructed image (*F*^–1^ *x*) was denoised by BM4D using adaptive noise level estimation with Rician noise distribution, while other parameters were kept at their default values. The denoised magnitude image was then multiplied by coil sensitivities, estimated from the coil calibration data using ESPIRiT (Uecker et al., 2014), to generate multi-coil complex data for the next iteration.

For the 20-direction 3T 0.53 mm protocol, NORDIC (Moeller et al., 2021) was used with *N_iter_*=2, *λ*_1_=10, and *λ*_2_=3. In each iteration, the multi-coil images *F*^–1^ *x* was coil-combined using the sensitivity map and then denoised by NORDIC (complex denoising), with the kernel size for g-factor map estimation set to 20×20×5, while other parameters remained at their default values. The denoised complex image was then multiplied by the sensitivity map to generate multi-coil data for the subsequent iteration. The PF data were reconstructed using P-LORAKS (Haldar and Zhuo, 2016).

The 1.22 mm data acquired at 3T were reconstructed using a standard SPIRiT with motion-induced phase error correction (Li et al., 2025). A joint SPIRiT reconstruction with distortion and phase error correction detailed in (Li et al., 2023) was used for reconstructing the 1.05 mm data acquired at 7T.

To demonstrate the benefit of DnSPIRiT in improving SNR while reducing blurring and bias typically introduced by denoising, fully sampled data from 3T 0.65 mm protocol were also reconstructed using SPIRiT followed by standalone BM4D denoising (denoted as “SPI-RiT+denoising”) with matched parameters with those used in DnSPIRiT. Results from SPIRiT, SPIRiT+denoising, and DnSPIRiT were compared for SNR, angular contrast-to-noise ratio (CNR), sharpness, and diffusion modelling bias. The noise standard deviation was estimated based on two repeated *b =* 0 acquisitions (Manzano Patron et al., 2024). The SNR for *b =* 0 and DWI images was computed by dividing mean signal with noise standard deviation within a white matter mask derived from the T1w image. Angular CNR was calculated as the ratio of the standard deviation of diffusion-weighted signal across six directions to the noise standard deviation within the white matter mask (Manzano Patron et al., 2024). The sharpness was quantified using the “Tenengrad” metric (Kecskemeti et al., 2018; Krotkov, 1988), defined as $\frac{1}{N}\sum_{i,j,k}\left(G_{x}{\left(i,j,k\right)}^{2}+G_{y}{\left(i,j,k\right)}^{2}+G_{z}{\left(i,j,k\right)}^{2}\right)$ where N denotes the voxel count, G_x,y,z_ is the spatial gradients along each axis, and i, j, k are spatial coordinates. To account for the impact of noise on spatial gradients, the Tenengrad values were normalized by the noise standard deviation of each method. To assess bias in diffusion model fitting, the 0.65 mm data from SPIRiT, SPIRiT+denoising, and DnSPIRiT as well as on 1.22 mm data of the same subject were fit to a diffusion tensor model (“dtifit”). Fractional anisotropy (FA) was computed within the T1w-derived white matter mask and mean diffusivity (MD) was computed within a brain mask. The higher-SNR, 24-direction 3D multi-slab 1.22 mm data served as the reference for evaluating bias in the 0.65 mm reconstructions. Additionally, we evaluated the similarity of FA and MD distributions between each high-resolution reconstruction and the 1.22 mm reference using the Kolmogorov-Smirnov (KS) statistic which quantifies the maximum difference between two cumulative distribution functions, with lower KS values indicating closer agreement with the reference.

To evaluate DnSPIRiT’s performance on under-sampled data, fully sampled 3T 0.65 mm data were retrospectively under-sampled by selecting 2 and 3 evenly spaced segments from the original 6 segments, corresponding to acceleration factors of *R_eff_*=3 and 2, respectively. The normalized root mean squared error (NRMSE) between the under-sampled and fully sampled data reconstructed using SPIRiT and DnSPIRiT was calculated within a brain mask to quantify their similarities.

### 2.4 Diffusion analyses

Image post-processing was conducted using the FMRIB Software Library (FSL) (Smith et al., 2004) unless indicated otherwise. Slab combination and correction for slab saturation artifacts were performed for the diffusion data using nonlinear inversion of slab profile encoding (NPEN) (Wu, Koopmans, et al., 2016). The images were corrected for Gibbs ringing (Bautista et al., 2021). A whole-brain field map was estimated using blip-reversed *b =* 0 image volumes using “topup” (Andersson et al., 2003). For the 6-direction diffusion data from 3T 0.65 mm protocol and 7T 0.61 mm protocol, data from different diffusion directions were aligned using “eddy_correct”, and “applytopup” was used to address susceptibility induced off-resonance distortions. For the 20-direction data from 3T 0.53 mm protocol, the diffusion data and the field map estimated by “topup” were input to “eddy” (Andersson and Sotiropoulos, 2016) to correct for off-resonance distortions, eddy current effects, and subject motion. The 1.22 mm and 1.05 mm data were also processed with NPEN, Gibbs ringing correction, “topup”, and “eddy”, followed by “flirt” for head position alignment with high-resolution data but without upsampling. The T1w image was corrected for bias field using “fast” and co-registered to the diffusion space by applying the inverse transform obtained from “*epi*_reg”. The co-registered T1w image was then processed by FreeSurfer’s “recon-all” (Fischl, 2012) to produce brain segmentations. All diffusion analyses were performed in the native diffusion space.

Diffusion tensor model fitting was performed on all diffusion data using “dtifit”. For the 20-direction 0.53 mm data and the 24-direction 1.22 mm data, fibre orientation distributions (FOD) were estimated voxel-wise using constrained spherical deconvolution (Tournier et al., 2004). A response function was first estimated with maximum harmonic degrees of 4 using MRtrix3’s “dwi2response” function and the “fa” algorithm (Tournier et al., 2019), followed by FOD estimation via MRtrix3’s “dwi2fod” function with the “csd” algorithm.

Tractography was performed to reconstruct three representative white matter tracts from the 0.53 mm and 1.22 mm datasets to evaluate the data quality. Specifically, the acoustic radiation, cingulate gyrus part of cingulum, and corticospinal tract were reconstructed with the seed, waypoint, target, and exclusion masks from “autoPtx” (De Groot et al., 2013). Tractography was executed with MRtrix3’s “tckgen” function using the “iFOD2” algorithm (Tournier et al., 2010), with the cutoff value set to 0.3, which specifies the minimum FOD amplitude required to continue tracking. For each tract, a matched number of streamlines were generated from the 0.53 mm and 1.22 mm resolution data.

To assess the “gyral bias” problem, we performed tractography within selected gyri to observe the distribution of streamline termination points. Masks were manually drawn to delineate several representative gyri on 0.53 mm and 1.22 mm data, and tractography was performed using these masks as seeds and masking region-of-interests (ROIs) using “tckgen” with “iFOD2” with a matched number of seeds and a minimum tract length of 5 mm.

For short association U-fiber comparison between the 0.53 mm and 1.22 mm data, tractography was performed to reconstruct U-fibers on both datasets. Supplementary Fig. 2 shows the seed, waypoint, and ROI masks for the tractography. Specifically, the seed mask was set to cortical gray matter, obtained by subtracting the white matter from the cortical ribbon mask segmented from the T1w image by FreeSurfer’s “recon-all” (Supplementary Fig. 2a). The waypoint mask was the white matter mask (Supplementary Fig. 2b) to ensure the fibers could not remain entirely in the gray matter. The ROI mask which defines the region in which tracts are displayed (i.e., streamlines exiting the mask will be truncated) was the cortical ribbon mask dilated using MRtrix3’s “maskfilter” with the dilation filter applied for four times (Supplementary Fig. 2c), excluding non-relevant deep white matter tracts. The tractography was performed using “tckgen” with “iFOD2” and a minimum track length of 10 mm. On the 0.53 mm data, one seed was generated from each cortical gray matter voxel and the cutoff value was set to 0.4. On the 1.22 mm data, two seeding mechanisms were evaluated: one seed per voxel (Song et al., 2014) and 12 seeds per voxel, with the latter compensating for the voxel count difference due to resolution and demonstrating the impact of an increased seed number. A higher FOD cutoff value of 0.7 was applied to the 1.22 mm data in both seeding strategies to account for the higher signal intensity and FOD amplitude at lower resolution, which helped reduce false-positive streamlines predominantly confined to gray matter. The 0.53 mm data (one seed per voxel) and 1.22 mm data (12 seeds per voxel) produced comparable streamline numbers (1.75 M and 1.84 M, respectively), while the 1.22 mm data with one seed per voxel yielded 0.15 M streamlines.

## 3 Results

**Fig. 1:**
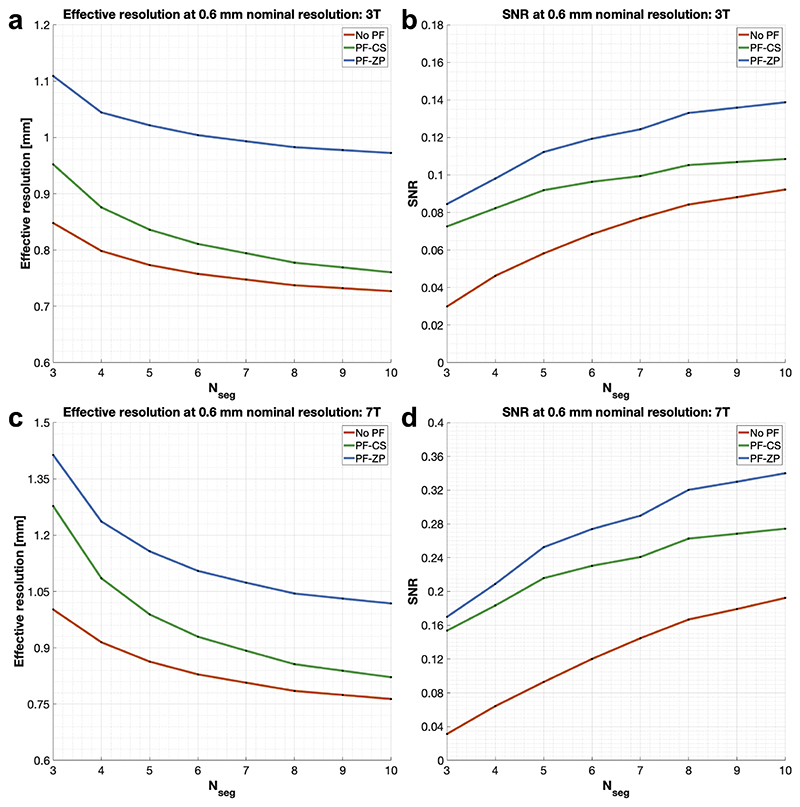
Simulation of effective resolution and SNR for 0.6 mm diffusion-weighted EPI. Three sampling strategies are evaluated: no partial Fourier (No PF, red), 6/8 partial Fourier with conjugate symmetric filling (PF-CS, green), and 6/8 partial Fourier with zero padding (PF-ZP, blue). Simulations are conducted for 0.6 mm diffusion-weighted EPI at 3T (a, b) and 7T (c, d) for white matter, using TR=2.5 s, b-value=1000 s/mm^2^, bandwidth=992 Hz/pixel, and tissue relaxation parameters T1/T2/T2*=832/79.6/53.2 ms (3T) and 1220/47/26.8 ms (7T) for different in-plane segmentation numbers (*N_seg_*). SNR is computed based on the field strength, bandwidth, number of phase-encoding lines and partitions, simulated effective voxel size, TE, and TR.

Simulation results for effective resolution and SNR across different in-plane segmentation numbers (*N_seg_*) for 0.6 mm EPI-based dMRI acquisitions at 3T and 7T are shown in Fig. 1. In all cases, T2* blurring reduces the effective resolution, but increasing *N_seg_* helps mitigate this effect (Fig. 1a, c). PF acquisitions can improve SNR by shortening the TE, but they introduce additional blurring, particularly when zero-padding (ZP) reconstruction is used. Although conjugate symmetry (CS) methods theoretically mitigate PF-induced blurring, in practice, phase errors lead to imperfect symmetry, leading to a compromise in effective resolution compared to full k-space sampling. These findings emphasize the importance of using a fair number of *N_seg_* to achieve higher effective resolution, especially for submillimeter imaging which incurs heavy T2* blurring due to the long EPI readout trains. Additionally, the simulations show that increasing *N_seg_* improves SNR due to a shorter TE (Fig. 1b, d). At 7T, the shorter T2* exacerbates blurring, necessitating a higher *N_seg_* to reduce the readout duration and maintain resolution comparable to that at 3T (Fig. 1a, c). Furthermore, the shorter T2 at 7T requires a larger *N_seg_* to reduce TE and better leverage the SNR benefit from the higher field strength (Fig. 1b, d). Based on these findings, we selected *N_seg_*= 6 for the 3T protocols and *N_seg_*= 8 for the 7T protocol to balance scan time, effective resolution, and SNR. The simulations suggest that our three high-resolution protocols – 3T 0.65 mm (*N_seg_*= 6, no PF), 3T 0.53 mm (*N_seg_*= 6, with PF=3/4), and 7T 0.61 mm (*N_seg_*= 8, no PF) – achieve similar effective resolutions along the phase-encoding direction of approximately 0.8 mm. Notably, the SNR for 0.6 mm acquisitions is considerably lower than for 1 mm acquisitions, with a nearly threefold difference (Supplementary Fig. 3), highlighting the substantial SNR challenges in achieving high-resolution dMRI and the critical need for higher *N_seg_* to compensate for SNR loss.

**Fig. 2:**
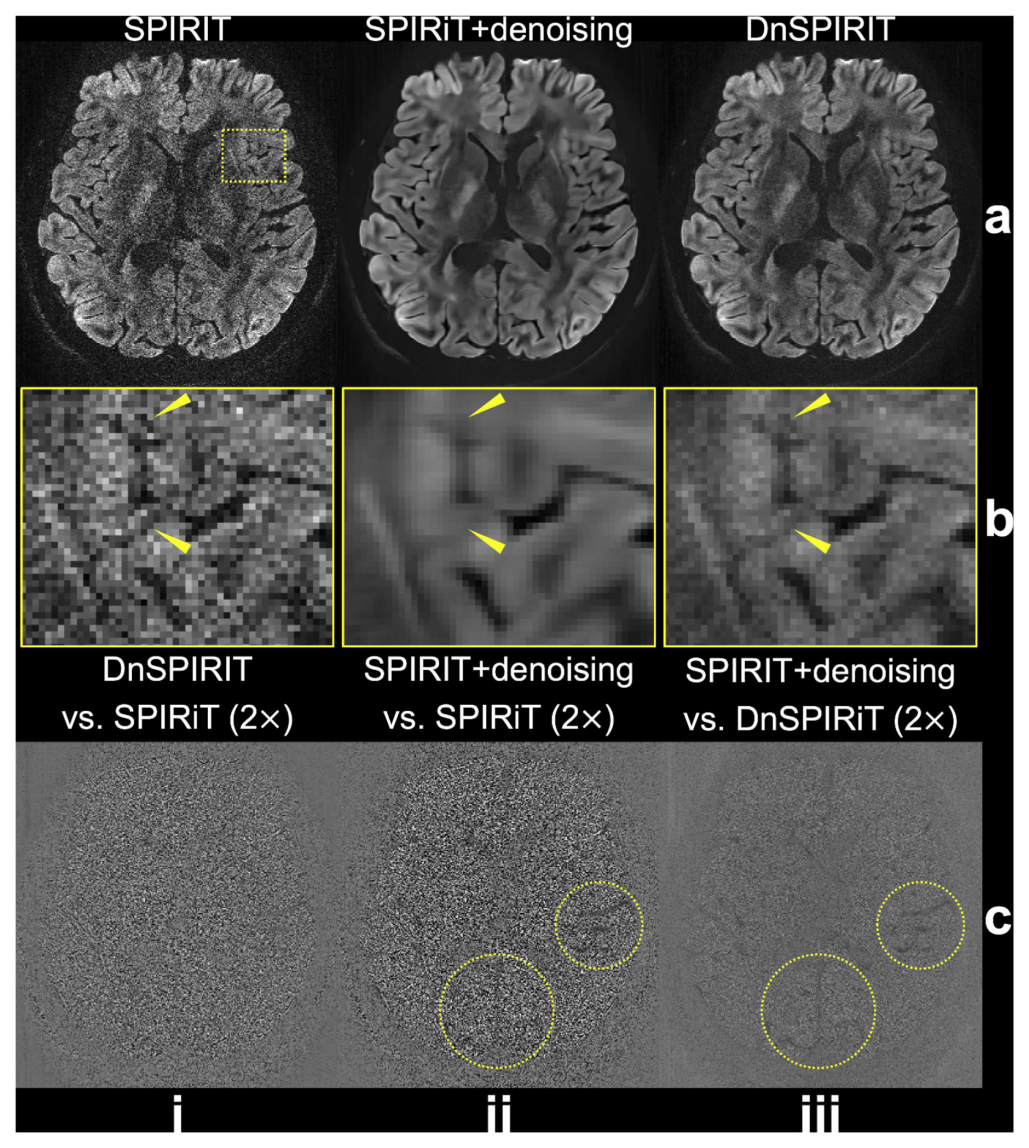
Comparison of image reconstruction and processing strategies. In-vivo diffusion-weighted data (*b* = 1000 s/mm^2^) from 3T 0.65 mm protocol along (–0.45, –0.83, 0.32) are reconstructed using SPIRiT (a, i), SPIRiT followed by standalone BM4D denoising (SPIRiT+denoising, a, ii), and denoiser-regularized SPIRiT (DnSPIRiT, a, iii), with an enlarged region showing the image detail (b). Yellow arrows indicate regions where anatomical structures appear blurred in SPI-RiT+denoising but are better preserved by DnSPIRiT. The difference maps between DnSPIRiT and SPIRiT (c, i), SPIRiT+denoising and SPIRiT (c, ii), SPI-RiT+denoising and DnSPIRiT are also shown to illustrate the distribution of noise and structural differences (highlighted by yellow circles) across methods.

Fig. 2 demonstrates image results from different reconstruction and processing strategies using fully sampled data from the 3T 0.65 mm protocol. Our acquisition scheme produces high-resolution diffusion images with crisp details and adequate SNR even when using the standard SPIRiT reconstruction (Fig. 2a, b, i). The lower noise variance outside the brain is likely attributed to SPIRiT’s inherent noise suppression, as the self-consistency constraint $(\lambda_{1}{\left\|(G-I)x\right\|}_{2}^{2}$ in Eq. (3)) enforces consistency with the calibration data containing only meaningful signals. This effectively suppresses background noise that is uncorrelated across coils in regions lacking signal, such as areas outside the brain (Lustig and Pauly, 2010; Murphy et al., 2012). Applying BM4D denoising to SPIRiT results (SPIRiT+denoising) substantially enhances the SNR but also introduces notable image blurring, obscuring fine anatomical details (yellow arrows, Fig. 2a, b, ii). In contrast, the proposed DnSPIRiT method retains the SNR improvement of denoising while effectively reducing the blurring, thereby better preserving anatomical structures (yellow arrows, Fig. 2a, b, iii). The difference map between SPIRiT and DnSPIRiT primarily reflects noise without noticeable anatomical structures or biases (Fig. 2c, i). However, the difference between SPIRiT and SPIRiT+denoising (Fig. 2c, ii) reveals residual anatomical structures, particularly in large sulci located in the left and posterior brain regions (yellow circles in Fig. 2c), indicating that the standalone denoising introduces structural changes. To verify that DnSPIRiT corrects these discrepancies rather than simply reintroducing noise, we show the difference between DnSPIRiT and SPIRiT+denoising (Fig. 2c, iii), which highlights DnSPIRiT’s ability to reduce denoising-induced structural artifacts, likely due to its improved data fidelity. The image reconstruction results of three other diffusion encoding directions from SPIRiT and DnSPIRiT are shown in Supplementary Fig. 4, demonstrating consistent performance with results in Fig. 2.

The quantitative comparisons of these reconstruction strategies are shown in Table 2. Consistent with observations in Fig. 2 and prior findings (Manzano Patron et al., 2024), SPIRiT+denoising yields a ~100 % increase in SNR compared to SPIRiT alone. This noise suppression also improves angular CNR by 34 %, and results in lower DTI bias, especially in FA, relative to the 1.22 mm reference. However, this comes at the cost of a 44 % reduction in image sharpness due to blurring introduced during post hoc denoising. In contrast, DnSPIRiT delivers a balanced performance: while SNR and CNR are slightly lower than SPIRiT+denoising, they remain substantially higher than SPIRiT (~66 % higher SNR and 20 % higher CNR). At the same time, DnSPIRiT reduces image blurring by approximately half (only 22 % loss in sharpness vs. 44 % with SPIRiT+denoising). Importantly, DnSPIRiT achieves the most consistent FA and MD with 1.22 mm reference data, indicating effective bias mitigation. This improved quantitative accuracy is further supported by the FA and MD distribution comparisons (Supplementary Fig. 5), where DnSPIRiT exhibits the lowest Kolmogorov-Smirnov statistics for both FA (0.3335 for SPIRiT, 0.0524 for SPIRiT+denoising, and 0.0339 for DnSPIRiT) and MD (0.0670 for SPIRiT, 0.0673 for SPIRiT+denoising, and 0.0618 for DnSPIRiT), indicating the closest agreement with the reference distributions. Detailed SNR and sharpness values for each DWI volume are provided in Supplementary Tables 2 and 3, demonstrating consistent performance across all diffusion directions.

**Fig. 3:**
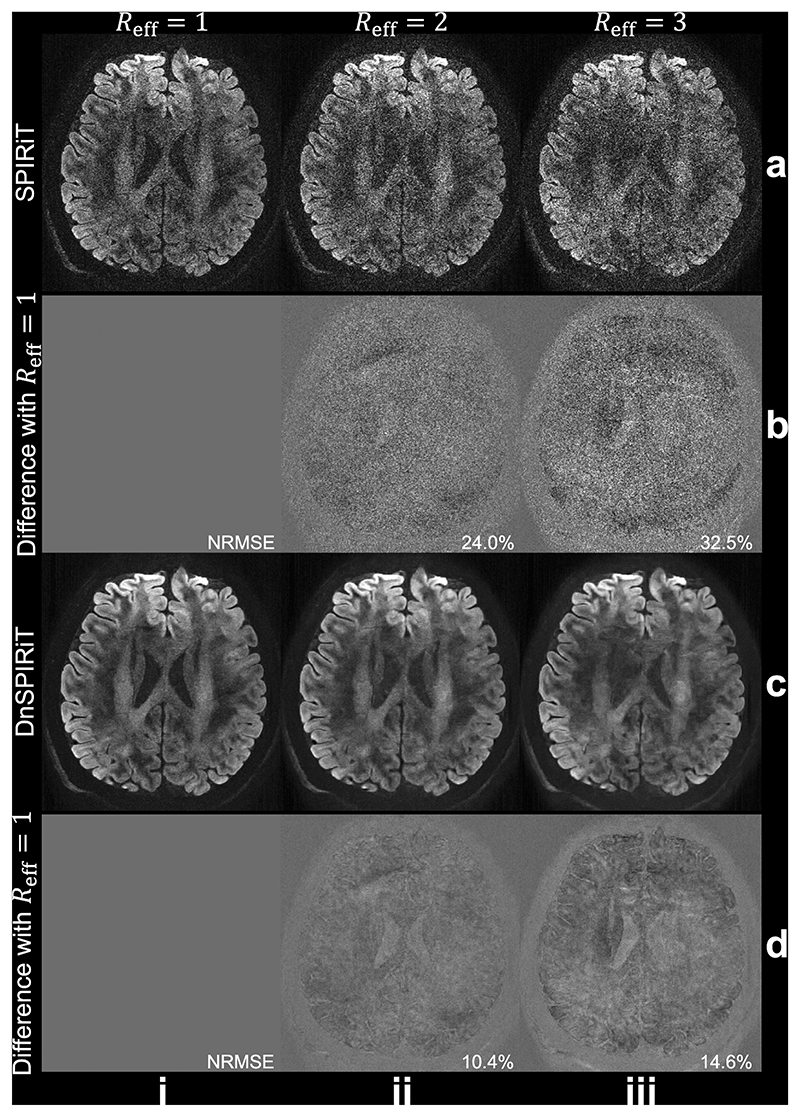
Retrospective under-sampled reconstruction. Retrospective under-sampling is applied to the fully sampled data (i) from 3T 0.65 mm protocol by selecting 3 segments (*R_eff_*=2, ii) and 2 segments (*R_eff_*=3, iii) from the total of 6 segments. The diffusion-weighted images along direction (−0.45, −0.86, −0.23) reconstructed using SPIRiT (a) and DnSPIRiT (c) and their difference with fully sampled reference (b, d) are shown to demonstrate the under-sampled reconstruction fidelity. The normalized root mean squared errors (NRMSE) between the under-sampled reconstructed image and the fully sampled reference of the whole slab are calculated within a brain mask to quantify their similarity.

The benefit of DnSPIRiT is also demonstrated in under-sampled reconstructions (Fig. 3). The standard SPIRiT reconstruction shows substantial degradation as the effective acceleration *R_eff_* increases, resulting in higher noise amplification and structured artifacts (Fig. 3a, b). In contrast, DnSPIRiT significantly mitigates these effects (Fig. 3c, d), leading to markedly lower NRMSE when compared to the fully sampled reference. It is worth noting that the NRMSE values reflect how each reconstruction degrades with acceleration relative to its corresponding unaccelerated reconstruction (i.e., *R* = 1 SPIRiT vs. *R* > 1 SPIRiT, or *R* = 1 DnSPIRiT vs. *R* > 1 DnSPIRiT), rather than the absolute SNR. At *R_eff_* >2 (Fig. 3, iii), even DnSPIRiT reconstruction exhibits visible anatomical errors, likely due to the intrinsic loss of SNR by $\sqrt{R_{eff}}$ and additional noise amplification caused by g-factor penalties. Therefore, we selected *R_eff_* =2 for the 3T 0.53 mm protocol to balance acquisition time with reconstruction accuracy, accepting some localized reconstruction bias, which primarily manifests in the ventricles (Fig. 3d, ii). Finally, it is worth noting that the retrospectively under-sampled acquisition used in the simulations may be more susceptible to motion artifacts than prospective under-sampling, due to the longer scan times of the fully sampled reference dataset.

**Fig. 4:**
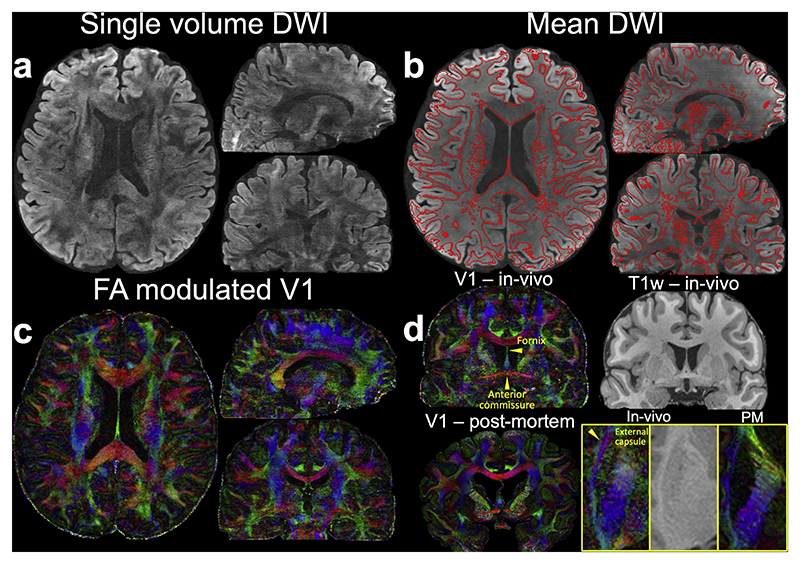
Data from 3T 0.65 mm protocol. (a) In-vivo diffusion data (*b* = 1000 s/mm^2^) including diffusion-weighted image (DWI) along (–0.45, 0.83, –0.32), (b) 6-direction mean DWI with gray-white matter boundary derived from the T1w image using overlayed, (c) fractional anisotropy (FA) modulated V1 at 0.65 mm isotropic resolution, (d) a representative sagittal view of the FA modulated V1 (V1 – in-vivo) alongside the T1-weighted image (T1w – in-vivo), highlighting key structures including the fornix, external capsule, and anterior commissure are presented. For comparison, post-mortem (PM) data (V1 – post-mortem) from previous studies (Tendler et al., 2022) are shown. Enlarged views of the external capsule from an axial plane are provided for both in-vivo and post-mortem data (d).

Fig. 4 depicts several visualizations of 3T 0.65 mm data on all three axes using the fully sampled data and DnSPIRIT reconstruction. The DTI-derived principal diffusion directions exhibit sharp details and high SNR even with only 6 diffusion encoding directions. The short effective echo spacing (0.21 ms), achieved through in-plane segmentation, results in good agreement of the mean diffusion-weighted image with the T1w-derived gray-white matter boundaries, indicating good anatomical fidelity (Fig. 4b). Small white matter tracts such as the fornix, external capsule, and anterior commissure are clearly visible on the 0.65 mm data with excellent anatomical fidelity (Fig. 4d). Moreover, these structures align well with existing post-mortem data at 0.5 mm resolution (Foxley et al., 2014; Tendler et al., 2022) (Fig. 4d).

**Fig. 5:**
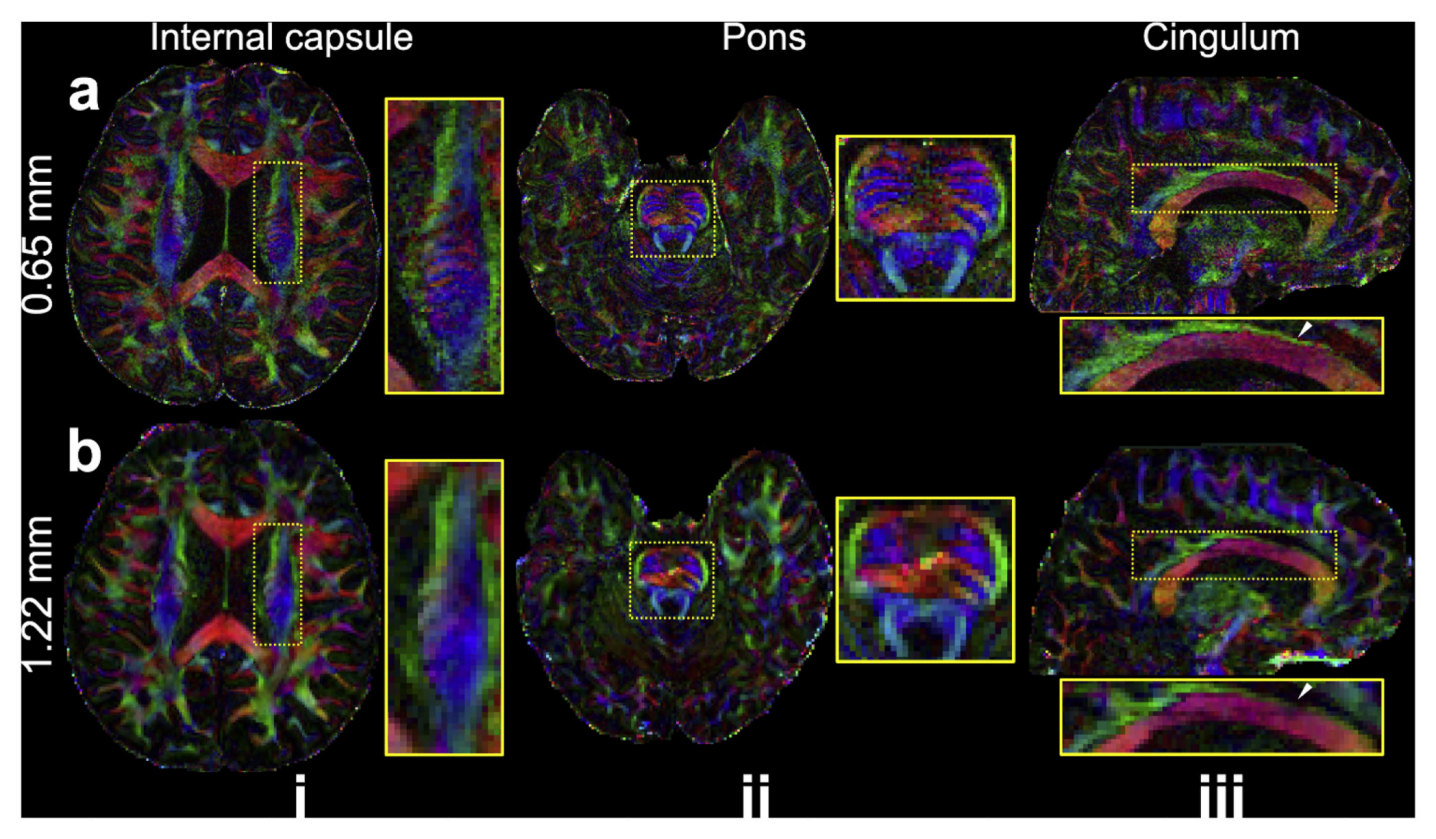
Comparison of 3T 0.65 mm and 1.22 mm isotropic resolution DTI. Two axial slices showing the internal capsule (i) and pons (ii), and a sagittal slice showing the cingulum bundle (iii) of FA modulated V1 of 0.65 mm (a) and 1.22 mm (b) isotropic resolution in-vivo diffusion data acquired at 3T are demonstrated. White arrows highlight the improved delineation of the cingulum bundle in the 0.65 mm data.

Compared to the conventional 1.22 mm resolution diffusion data, data from 3T 0.65 mm protocol reveals substantially finer structure details (Fig. 5). This includes the improved visualization of the striations through the internal capsule (Fig. 5, i), the interdigitating transverse pontine fibers (Fig. 5, ii), and the clearer delineation of the cingulum bundle (Fig. 5, iii).

**Fig. 6:**
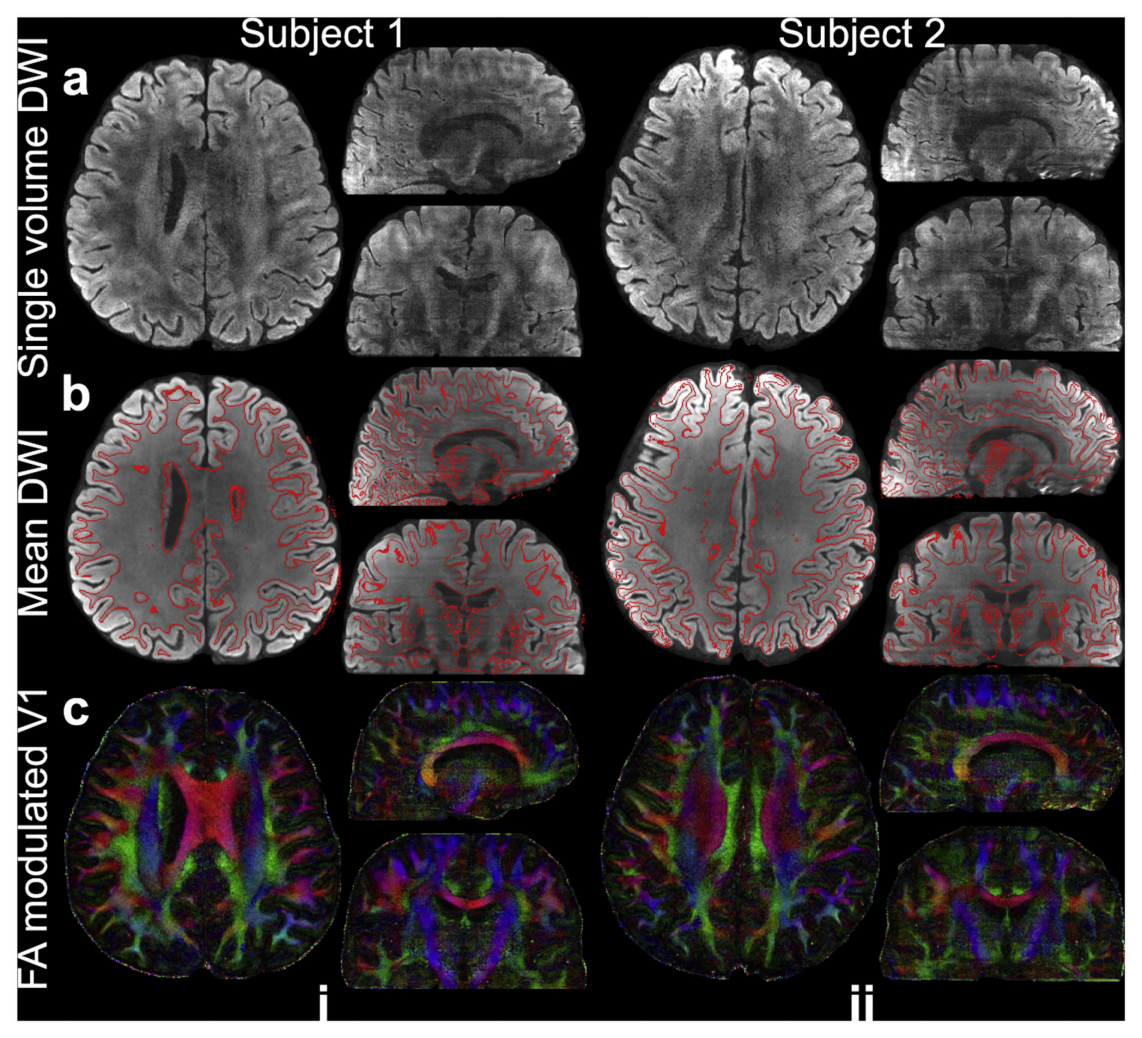
Data from 3T 0.53 mm protocol. (a) In-vivo diffusion data of two subjects (*b* = 1000 s/mm^2^) including diffusion-weighted image (DWI) along (0.5, –0.86, 0.02), (b) 20-direction mean DWI with gray-white matter boundary derived from the T1w image using FSL’s “fast” overlayed, (c) FA modulated V1 at 0.53 mm isotropic resolution.

Fig. 6 shows the high-resolution diffusion data from 3T 0.53 mm protocol. Across both subjects, our proposed method consistently achieves high image quality at 0.53 mm isotropic resolution with adequate SNR (Fig. 6a, c) and excellent agreement with anatomical boundaries derived from T1w data (Fig. 6b). Nevertheless, residual slab boundary artifacts remain visible in the 0.53 mm data, even after NPEN corrections. This is likely due to the extremely low SNR at the slab boundary slices, posing challenges for accurate slab profile estimation in NPEN.

**Fig. 7:**
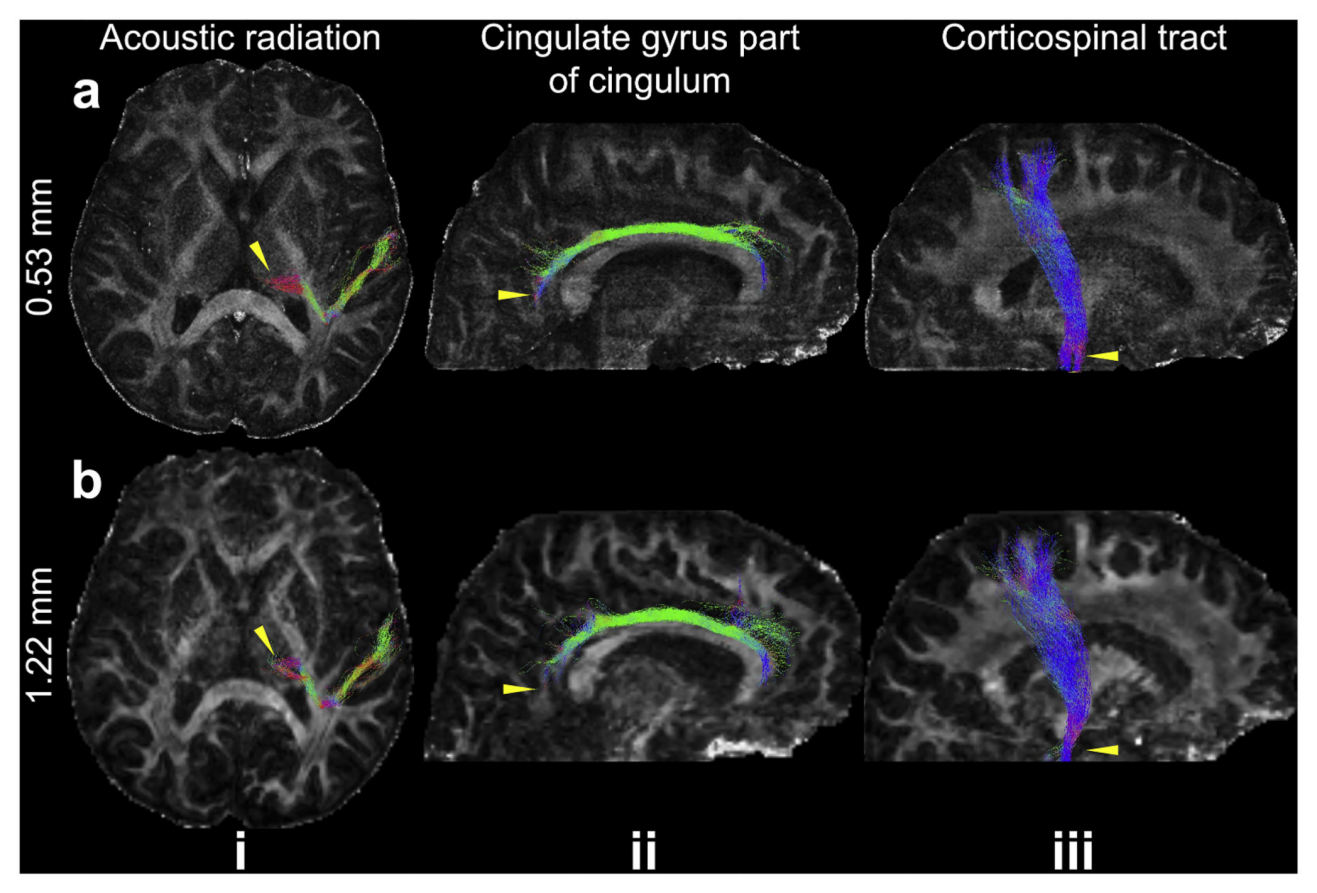
Example white matter tracts from the 0.53 mm and 1.22 mm data. The maximum intensity projections of three representative white matter tracts including acoustic radiation (i), cingulate gyrus part of cingulum (ii), and corticospinal tract (iii) are overlayed on the fractional anisotropy maps of 0.53 mm (a) and 1.22 mm (b) datasets. The yellow arrows indicate the region where the tractography on 0.53 mm data shows improvement compared to that on 1.22 mm data.

The high quality of the 0.53 mm data is further demonstrated through the successful reconstruction of three representative white matter tracts with distinct orientations and complex fiber architectures (Fig. 7). Specifically, our data enable the tracking of the acoustic radiation, which crosses several major brain fiber systems with complex fiber crossings (e.g., posterior limb of the internal capsule, corona radiata), making it difficult to reconstruct from in-vivo data (Maffei et al., 2018). On the 0.53 mm data, the acoustic radiation is successfully reconstructed and demonstrates a clearer termination in the thalamus with a well-defined curved trajectory compared to the 1.22 mm data (Fig. 7, i, yellow arrows). The cingulate gyrus portion of the cingulum, which predominantly follows an anterior-posterior trajectory, is also successfully reconstructed in both datasets. The tract appears more extensive in the retrosplenial region in the 0.53 mm data, likely due to the higher resolution (Fig. 7, ii, yellow arrows). For the corticospinal tract, which primarily runs superior-inferior, the 0.53 mm data reveal a thicker and more robust appearance near the pons (Fig. 7, iii, yellow arrows), which is likely due to reduced distortion and better anatomical fidelity in the 0.53 mm data compared to the 1.22 mm data (Supplementary Fig. 6). Notably, the tractography utilized masks from FSL’s “autoPtx”, which is designed for conventional resolution fiber tracking (i.e., 1–2 mm).

**Fig. 8:**
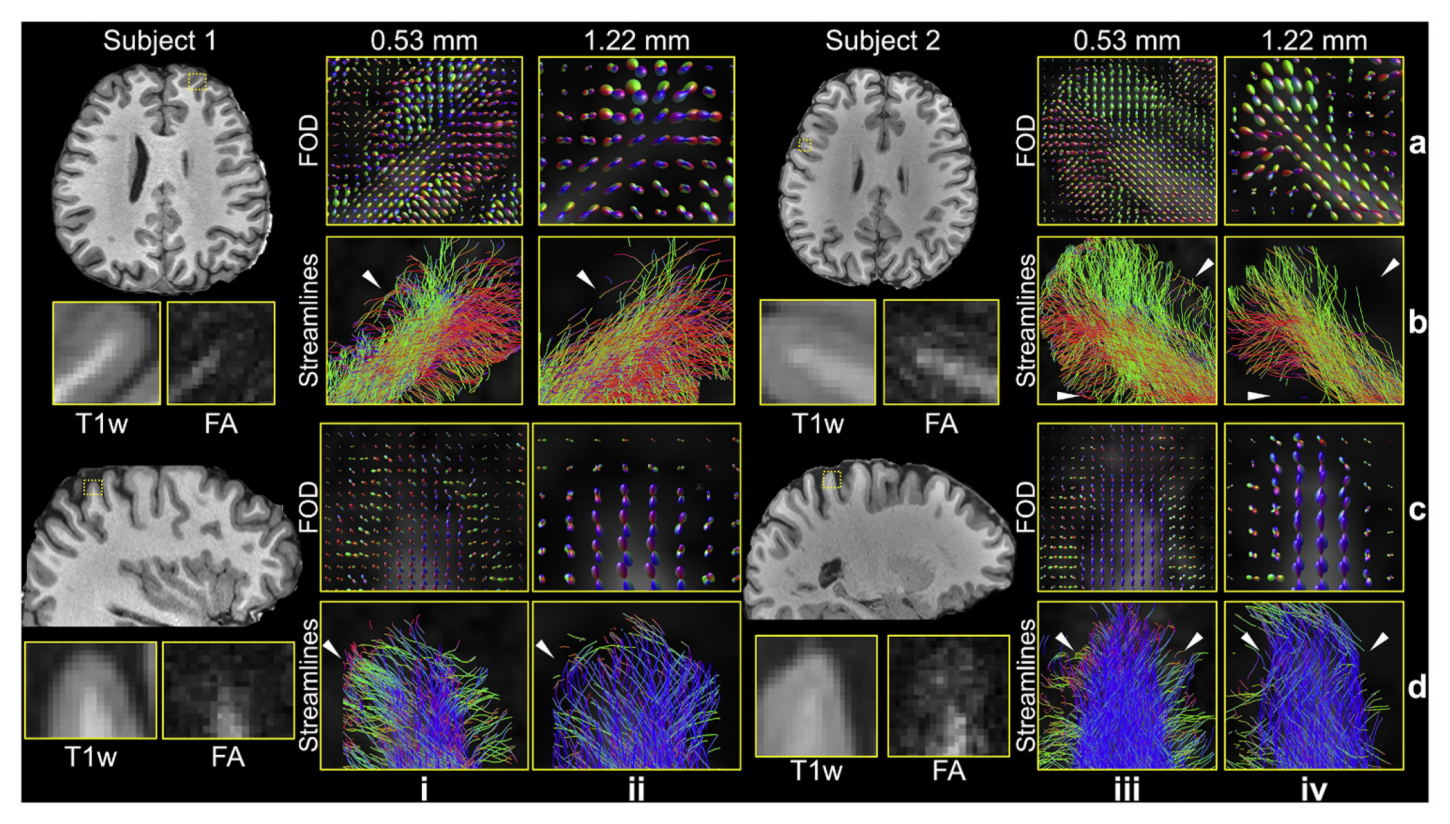
Gyral bias comparison between 0.53 mm and 1.22 mm data. The fiber orientation distributions (FOD) (a, c) and tractography streamlines (b, d) for representative gyri from the 0.53 mm (i, iii) and 1.22 mm (ii, iv) data of two subjects are shown to demonstrate the reduced gyral bias on high-resolution data (highlighted by white arrows). Co-registered T1w images, along with enlarged T1w and fractional anisotropy (FA) maps (0.53 mm isotropic resolution) for the selected gyri, are provided as anatomical references to aid visualization of the cortical structure.

The 0.53 mm data also effectively reduce the gyral bias problem, particularly in small gyri (Fig. 8). In the 1.22 mm data, gyral bias is evident, with streamlines preferentially terminating at the gyral crowns while fewer reach the gyral walls (Fig. 8, ii, iv, white arrows). In contrast, the 0.53 mm data show the expected fanning pattern, with a greater number of streamlines extending to the gyral walls (Fig. 8, i, iii, white arrows). This more even distribution of streamline termination points between gyral crowns and walls in the 0.53 mm data indicates a significant reduction in gyral bias with higher spatial resolution, consistent with previous post-mortem findings (Van Essen et al., 2014). Interestingly, we found the gyral bias problem in our data is less pronounced in larger gyri, where the expected fanning pattern is also visible in the 1.22 mm data (Supplementary Fig. 7). This observation is consistent with prior studies, which also demonstrated the fanning pattern in large gyri using 7T HCP data at 1.05 mm resolution (Gulban et al., 2018).

**Fig. 9:**
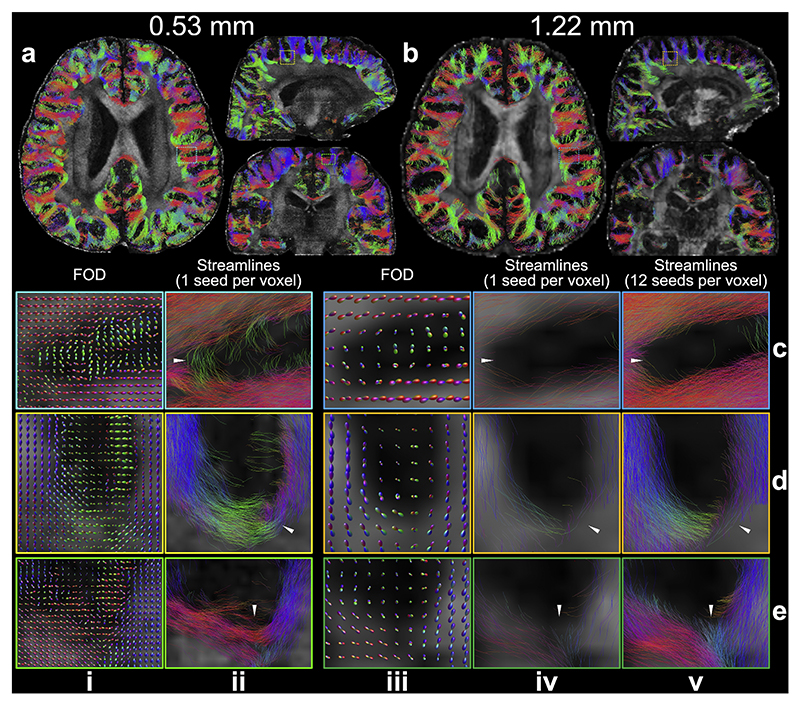
U-fibers comparison between 0.53 mm and 1.22 mm data. The whole-brain short association fibers tracked with one seed per voxel for 0.53 mm (a) and 1.22 mm (b) data are overlayed on fractional anisotropic maps. Representative enlarged regions (c-e) show the fiber orientation distributions (FOD) (i, iii), the tracked streamlines with one seed per voxel (ii, iv) for the 0.53 mm (i, ii) and 1.22 mm (iii, iv) data, and the tracked streamlines with 12 seeds per voxel for the 1.22 mm data (v) to compensate the difference in voxel numbers due to resolution. The white arrows indicate the region where the U-fibers on 0.53 mm data are better resolved at sharp turnings compared to that on 1.22 mm data.

Compared to the 1.22 mm data, the 0.53 mm data also provide an improved mapping of U-fibers (Fig. 9). Our tractography setup successfully identified short association fibers in both datasets (Fig. 9a, b). When using one seed per voxel for both, the 0.53 mm data reveal denser whole-brain short association fibers, likely due to the substantially increased voxel number (and therefore number of streamline seeds). These findings are consistent with previous studies (Song et al., 2014). Remarkably, the 0.53 mm data is able to resolve U-fibers even at sharp turnings along the gray-white matter boundary (Fig. 9c-e, i, ii). In contrast, even with increased seed numbers to compensate voxel number difference, the 1.22 mm data often fail to capture these curved trajectories, resulting in streamlines that continue along their original direction without arcing around the gyrus (Fig. 9, white arrows). The tractography on 0.53 mm data with one seed per voxel and that on 1.22 mm data with 12 seeds per voxel produced comparable numbers of streamlines (1.75 M vs. 1.84 M). This confirms that the improved tractography using the 0.53 mm data originates from more accurate estimation of fiber orientation and thus better capture of fiber curvature due to higher spatial resolution, rather than simply an increase in seed or streamline numbers.

**Fig. 10:**
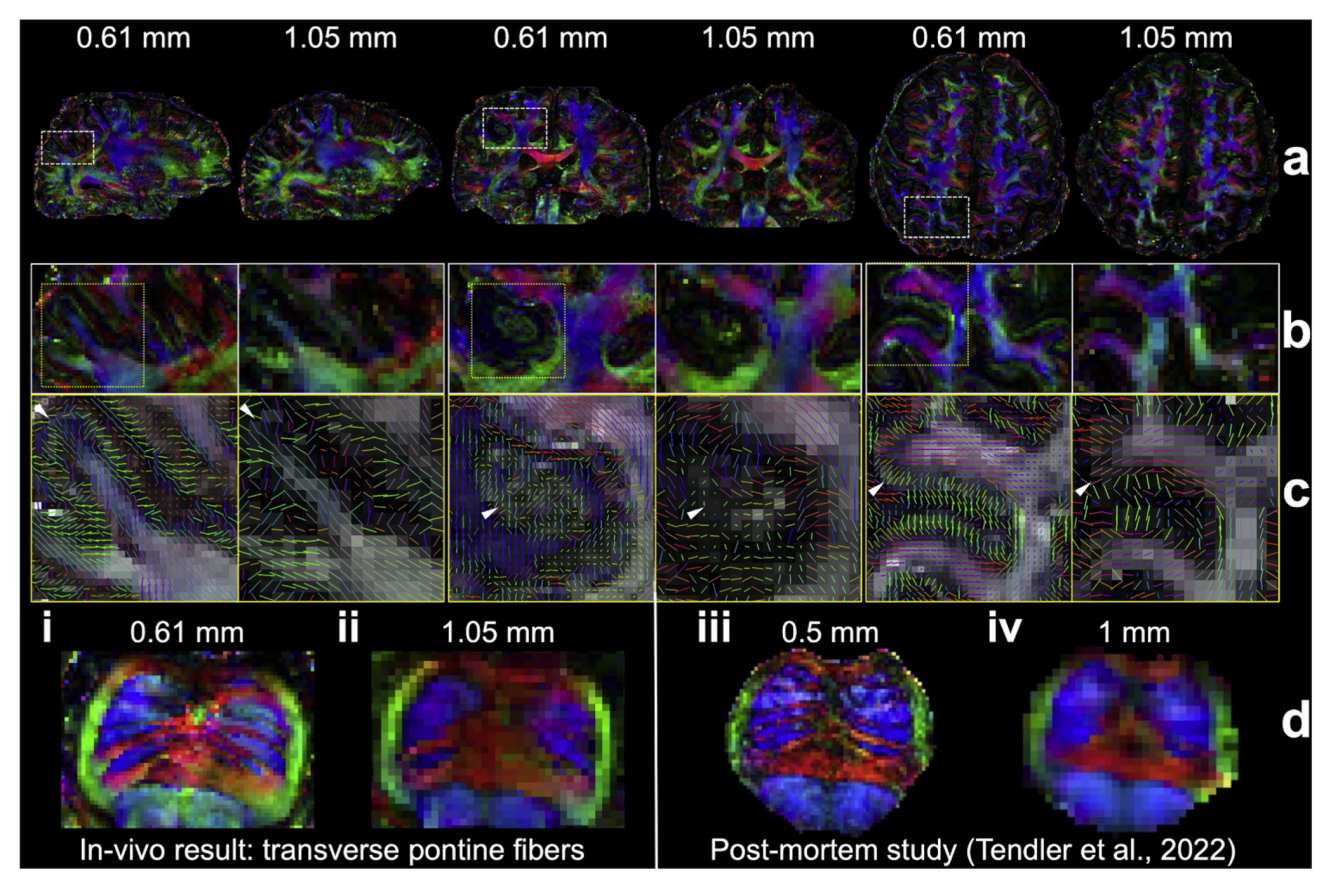
Comparisons of 7T 0.61 mm and 1.05 mm diffusion data. The sagittal, coronal, and axial slices of fractional anisotropy (FA) modulated V1 (a) and their enlarged regions (b, c) of 0.61 mm and 1.05 mm isotropic resolutions in-vivo diffusion data acquired at 7T are demonstrated, with white arrows highlighting the more detailed microstructure resolved by the high resolution at 0.61 mm. The traverse pontine fibers (d, i, ii) demonstrate similar patterns compared to previous post-mortem study (d, iii, iv) (Tendler et al., 2022) at 0.5 mm isotropic resolution from the same scanner.

Our acquisition and reconstruction framework shows robustness at an ultrahigh field of 7T (Fig. 10). The diffusion data from the 7T 0.61 mm protocol exhibit excellent SNR even with only six diffusion encoding directions, thanks to the SNR benefit of 7T (Fig. 1). Compared to the 1.05 mm isotropic resolution data, the submillimeter dataset reveals significantly better fanning patterns in the gyri and clearer delineation of small fiber structures (Fig. 10c, white arrows). Notably, our in-vivo results for resolving transverse pontine fibers (Fig. 10d, i, ii) are in excellent agreement with previous post-mortem data (Fig. 10d, iii, iv) acquired with much longer scan times (~20 h) and a distortion-free diffusion-weighted steady-state free precession (DW-SSFP) acquisition on the same 7T scanner (Foxley et al., 2014; Tendler et al., 2022). This consistency refers to the emergence of the same anatomical features, such as well-defined mediolateral pontine fibers, across in-vivo and post-mortem data at similar spatial resolutions. Given the high SNR and minimal blurring of post-mortem DW-SSFP imaging, the ability to resolve comparable anatomical detail in vivo highlights the quality of our submillimeter diffusion data.

## 4 Discussion

This study introduces an acquisition and reconstruction framework to achieve high-quality submillimeter dMRI. Our acquisition takes advantage of the optimal SNR efficiency provided by 3D multi-slab EPI, with in-plane segmentations integrated to shorten the effective echo spacing, readout, and TE for reduced distortion, T2* blurring, and SNR penalty. The sampling order is designed for improved robustness against subject motion. A denoiser-regularized reconstruction approach is proposed to suppress noise while maintaining consistency with the acquired data to minimize denoiser-induced bias or blurring. Our in-vivo experiments at 3T demonstrate that our method consistently produces high-quality diffusion data at 0.65 mm and 0.53 mm isotropic resolutions. The analyses of these submillimeter datasets reveal finer fiber architectures, mitigating the “gyral bias” problem and improving the precision of U-fiber tracking compared to data acquired at conventional resolutions of 1.22 mm from the same subjects at the same scanner. The framework also proves effective at 7T, delivering similarly robust results. These findings suggest that the proposed framework represents a promising advance in high-resolution dMRI, offering the potential for more accurate characterization of brain connectivity and novel applications in neuroscience research.

The optimization of our sampling strategies considers SNR, effective resolution, distortion, scan time, and motion robustness. Compared to 2D acquisitions, our segmented 3D multi-slab imaging offers superior SNR efficiency primarily due to the shorter TR achievable with 3D encoding. This advantage is demonstrated by the substantially higher SNR in our segmented 3D acquisition relative to a Siemens product 2D readout-segmented EPI (rs-EPI) acquisition with longer scan time and lower resolution (Supplementary Fig. 8). Previous studies achieved submillimeter dMRI primarily using 2D super-resolution methods such as gSlider (Liao et al., 2023; Setsompop et al., 2018). It is worth noting that 3D multi-slab imaging and gSlider share similar conceptual frameworks, where multiple contiguous slabs are excited and spatially encoded along the slice direction. The key distinctions are twofold: (i) 3D multi-slab employs Fourier encoding along the slice dimension, resulting in orthogonal encoding bases and high-fidelity voxel shapes, whereas gSlider relies primarily on RF-based encoding; (ii) 3D multi-slab typically uses thicker slabs with more extensive kz-encoding, intrinsically more compatible with shorter TRs and thus more SNR-efficient acquisitions for spin echo-based dMRI (Engström et al., 2015).

We integrated in-plane segmentation into 3D multi-slab imaging to further improve the data quality. For EPI-based sampling that is commonly used in both previous super-resolution methods and 3D multi-slab imaging, higher resolution imaging requires longer echo spacing, readout, and TE, and therefore more severe distortion, T2* blurring, and T2 signal decay. For example, at 3T with *N_seg_* *=* 3 sampling and without PF, assuming a 50 Hz B0 offset, for 0.6 mm imaging, the image displacement is 4.4 mm, image blurring (defined as (effective resolution-nominal resolution)/nominal resolution) is 41.3 %, and T2 signal decay (*e*^-*TE*/*T*2^) reduces the signal at TE to 10.3 %. In comparison, for 1 mm imaging, the corresponding values are 3.2 mm, 25.3 %, and 29.3 % (Supplementary Fig. 3). This means a larger *N_seg_* is required for submillimeter imaging to achieve high effective resolution, sufficient SNR, and reduced distortion, though at the cost of longer scan times. In the 3T 0.65 mm protocol, we acquired fully sampled data with *N_seg_*=6 without using PF, which achieved adequate SNR, high image sharpness, and excellent anatomical fidelity (Fig. 4). To acquire more diffusion encoding directions (e.g., to enable tractography), we explored the potential for acceleration by acquiring fewer segments (Fig. 3), using *R_eff_*=2 in the 3T 0.53 mm protocol without significantly compromising the image quality. This approach enabled the acquisition of 20 diffusion encoding directions in a one-hour session. Notably, the TR of 2 s in this protocol is within the optimal SNR-efficiency range. Additionally, we applied a sampling order that is robust to motion for 3D EPI, which particularly improves the *b =* 0 images where motion artifacts are more noticeable due to strong CSF signal aliasing (Supplementary Fig. 1).

Previous studies have demonstrated segmented 3D multi-slab dMRI acquisitions such as 3D readout-segmented EPI (rs-EPI) (Frost et al., 2014), 3D MUSE (Chang et al., 2015), and 3D-MB-MUSE (Bruce et al., 2017). Our acquisition method differs from these in several key aspects. Instead of rs-EPI, we employed phase-encoding segmented EPI, which enables more flexible acceleration via shot under-sampling. Both 3D MUSE and 3D-MB-MUSE mitigated slab-boundary artifacts through multiple FOV-shifted acquisitions: 3D MUSE acquired three shifted slab volumes while 3D-MB-MUSE used separate odd and even slab acquisitions. This acquisition approach effectively reduced slab boundary artefacts, but also substantially prolonged total acquisition time. In contrast, we applied NPEN for slab-boundary correction without additional scan-time penalty. Importantly, compared to previous 3D multi-slab dMRI methods, our work achieves much higher spatial resolution (0.53 mm isotropic vs. 0.85–1.1 mm in prior studies) by using a larger number of segments (*N_seg_*=6 vs. *N_seg_* =4). Despite the increased resolution and number of segments, our overall scan efficiency was improved through shot under-sampling (*R_eff_* =2), resulting in comparable or shorter scan times (2.7 min/volume at 0.53 mm) relative to previous methods (rs-EPI: 2.6 min/volume for partial FOV at 1.1 mm; 3D MUSE: 7.2 min/volume at 0.85 mm; 3D-MB-MUSE: 2.4 min/volume at 1.0 mm with multiband=3).

Our denoiser-regularized reconstruction DnSPIRiT effectively suppresses noise while maintaining image fidelity by enforcing consistency with the acquired data. While the idea of integrating denoising as a form of image-domain prior is conceptually aligned with earlier submillimeter dMRI reconstruction methods, such as those developed for gSlider (Haldar et al., 2020; Ramos-Llorden et al., 2020), DnSPIRiT explicitly leverages the SNR benefit provided by denoisers with a flexible integration. Specifically, we build upon SPIRiT, a model-based iterative k-space reconstruction framework (Lustig and Pauly, 2010). Compared to GRAPPA (Griswold et al., 2002), it makes more efficient use of all acquired k-space and allows flexible incorporation of regularizations; and compared to SENSE (Pruessmann et al., 2001, 1999), it offers better noise suppression and improved robustness, especially in regions where accurate explicit coil sensitivity maps are challenging to obtain (Hamilton et al., 2017). By integrating a denoiser into SPIRiT, we essentially use the denoised image as a regularization to suppress noise during reconstruction, rather than as a final output. Our experiments demonstrate that DnSPIRiT substantially improves SNR and angular CNR compared to SPIRiT, while reducing blurring and structural alterations observed with SPIRiT+denoising (Fig. 2, Table 2). Notably, DnSPIRiT also achieved the lowest bias in FA and MD relative to the 1.22 mm reference with lower resolution, more diffusion directions, and higher SNR, indicating enhanced fidelity in quantitative diffusion measurements. This denoiser integration is especially beneficial in under-sampled reconstructions, where inherent noise levels are higher (Fig. 3).

The plug-and-play nature of DnSPIRiT offers a flexible framework for incorporating advanced denoisers. While this approach does not inherently guarantee convexity, prior studies have demonstrated that it exhibits robust empirical convergence across a wide range of denoisers (Ahmad et al., 2020; Reehorst and Schniter, 2018). We demonstrated its compatibility with BM4D for 6-direction data denoising and NORDIC for 20-direction data denoising. As shown in Supplementary Fig. 9, DnSPIRiT demonstrates stable convergence even when used with BM4D, a nonlinear, non-differentiable denoiser with a non-convex gradient field (Reehorst and Schniter, 2018). Recent progress in image denoising (Fadnavis et al., 2020; Moran et al., 2020; Quan et al., 2020; Tian et al., 2020, 2022; Wu et al., 2025; Xiang et al., 2023) offers promising opportunities to further enhance DnSPIRiT’s performance. We adopted Self2Self (Quan et al., 2020) on our 3T 0.65 mm data, showing the efficacy of the deep learning-based denoiser in improving submillimeter dMRI’s SNR (Supplementary Fig. 10). While comprehensive tuning of deep learning architectures and full integration into the DnSPIRiT framework are beyond the scope of this study, these represent exciting directions for future research.

The in-vivo experiments comparing submillimeter data with prospectively acquired conventional-resolution data (1.22 mm and 1.05 mm) validate the benefits of higher spatial resolution for diffusion analyses. In previous studies (Chang et al., 2015; Song et al., 2014; Wang et al., 2021), the comparisons between the high-resolution and low-resolution data were based on retrospective under-sampling. One limitation of those comparisons is that the increased image blurring and SNR penalty associated with high-resolution imaging (Fig. 1, Supplementary Fig. 3) are retained in the under-sampled low-resolution data, leading to lower image quality than what can be practically achieved. Instead, we prospectively acquired data at 1.22 mm and 1.05 mm with practical imaging protocols from the same scanners as the submillimeter resolution data. Using this approach, the datasets at conventional resolutions also demonstrate excellent data quality. For instance, the expected fiber fanning pattern can be resolved at large gyri from the 1.22 mm data (Supplementary Fig. 7). These prospective acquisitions allow for a more realistic comparison and demonstrate that submillimeter data offer significant advantages over 1.22 mm data. Specifically, submillimeter data excel at resolving fine details, such as the small-scale striation patterns within the internal capsule and pons, which are characterized by complex crossing fibers (Fig. 5). They also reduce gyral bias in small gyri (Fig. 8) and improve the mapping of U-fibers with sharp turnings along the gray-white matter boundary (Fig. 9). Additionally, improvements were also observed in reconstructing representative white matter tracts (Fig. 7), where we explicitly did not deviate from conventional tractography methods for a fair comparison to the 1.22 mm data. It is expected that the advantage of submillimeter data would be even more pronounced with tailored tractography methods designed for such spatial resolution.

The tractography results highlight the benefits of higher spatial resolution for resolving both intra-gyral (gyral bias; Fig. 8) and intergyral (U-fibers; Fig. 9) fiber architectures. While increasing the b-value can enhance angular resolution and aid in resolving complex fiber crossings, it does not replicate the anatomical precision made possible by submillimeter voxel sizes. Angular and spatial resolutions are complementary: higher b-values improve sensitivity to fiber orientation, but accurately mapping fine anatomical structures, such as U-fibers and cortical terminations, requires sufficiently small voxel sizes (Calabrese et al., 2014). As shown in Figs. 8 and 9, at 3T, the 24-direction 1.22 mm data are capable of resolving major fiber crossings, but fail to capture sharp turnings and the detailed trajectories of fine fibers, primarily due to their limited spatial resolution. In contrast, the 20-direction 0.53 mm data achieve superior tractography performance. In the 7T data, 6-direction 0.61 mm acquisitions yield more biologically plausible fiber orientation patterns than the 48-direction 1.05 mm data (Fig. 10). These phenomena highlight that high spatial resolution plays a critical role in preserving fiber structural accuracy, an advantage that cannot be compensated for with extensive q-space sampling and high b-values at lower resolutions (Calabrese et al., 2014; Zhu et al., 2025). Future studies that systematically vary both spatial and angular resolution will be valuable for further quantifying this trade-off and guiding protocol optimization.

We implemented our sequence using Pulseq to improve the accessibility of our method. The scanner-agnostic, open-source nature of Pulseq facilitates the straightforward translation of our framework to different scanner platforms. Demonstrating the ability to produce high-quality data on a clinical scanner (Siemens 3T Prisma) highlights the potential for broader application of our methods across various clinical and research settings. We further demonstrated the method’s compatibility on a higher field strength at 7T (Siemens 7T Magnetom). Excitingly, the state-of-the-art high-performance scanners with stronger gradients (Feinberg et al., 2023; Foo et al., 2020; Huang et al., 2021; Ramos-Llordén et al., 2025; Setsompop et al., 2013), offer promising opportunities to further reduce readout durations and TE, improving image quality and potentially enabling even higher spatial resolutions.

Robust, high-quality submillimeter dMRI opens new opportunities for neuroscience and clinical research. In this study, we have demonstrated its ability to reduce gyral bias and improve U-fiber mapping. These advances hold promise in pre-surgical planning and disease research. Gyral bias limits the ability of tractography to resolve fibers terminating in sulcal walls, which can compromise cortical connectivity mapping and surgical targeting (Reveley et al., 2015; Schilling et al., 2018). Improved spatial resolution mitigates this issue, enabling more accurate localization of critical pathways for procedures such as tumor resection, epilepsy surgery, and deep brain stimulation (Kamagata et al., 2024). Enhanced U-fiber mapping is also valuable for studying neuro-development and neurodegeneration. Superficial white matter U-fibers form the majority of brain axonal connections and play a key role in cortico-cortical communication (Markov et al., 2013). Due to their late myelination and early vulnerability, they are among the first affected in neurodegenerative diseases such as Alzheimer’s (Carmeli et al., 2014; Fornari et al., 2012). Accurately imaging these fibers may aid early detection and improved understanding of disease progression.

One limitation of our method is the relatively long scan and reconstruction times. While hour-long sessions are common in research settings for submillimeter dMRI, for example, 96 min for 0.85 mm 12-direction 3D MUSE (Chang et al., 2015), 50 min for 0.66 mm 64-direction gSlider (Setsompop et al., 2018), 117 min for 0.6 mm 64-direction gSlider-BUDA (Liao et al., 2021), and 78 min for 0.5 mm 20-direction ROMER-EPTI (Dong et al., 2024), such durations can increase subject discomfort and motion, which pose challenges for clinical applications. To keep the total scan time within a ~1-hour session (65 min for the 3T 0.53 mm protocol), we also restricted the FOV along the slice direction to focus primarily on the cerebrum, without achieving full coverage of the cerebellum. Additionally, the current protocol includes only a single *b =* 1000 s/mm^2^ shell. Although higher b-values (e.g., *b =* 2000 s/mm^2^) and multi-shell acquisitions are beneficial for advanced tractography and microstructural modeling, the relatively long scan time per volume (2.7 min for the 3T 0.53 mm protocol) limits the number of diffusion directions that can be practically acquired. Further acceleration could be achieved by leveraging k-q space joint reconstructions (Mani et al., 2021; Wu et al., 2019), which shares information across diffusion directions. Additionally, recent advances in self-navigated 3D multi-slab EPI (Li et al., 2025) could be integrated into our framework to enhance scan efficiency by eliminating the navigator acquisition, which accounted for approximately 30 % of the total imaging time, without compromising SNR. Simultaneous multi-slab acquisitions also offer a promising approach for further shortening the TR and accelerating the scan (Dai et al., 2021, 2019). On the reconstruction side, processing submillimeter data remains time-intensive due to the large matrix size. For example, processing data from the 3T 0.53 mm protocol (414×414×27 per slab) takes approximately 12 h per slab on a single 2.9 GHz Quad-Core Intel Core i7 CPU. Model-based deep learning reconstruction methods (Aggarwal et al., 2018) present a promising avenue to significantly reduce computational time and potentially further improve SNR. However, these methods come with inherent challenges, including the need for high-quality training data and concerns about generalizability across datasets acquired using different protocols, which need to be carefully addressed.

Residual slab boundary artifacts remain an area for potential improvement in future work. Unlike previous studies that addressed these artifacts by acquiring multiple sets of images with shifted slab profiles at the cost of substantially longer scan time (Bruce et al., 2017; Chang et al., 2015), we employed NPEN for correction without additional time cost. NPEN demonstrated satisfactory performance for data from 3T 0.65 mm protocol (Fig. 4) and 7T 0.61 mm protocol (Fig. 10), but residual artifacts remain visible in the 3T 0.53 mm data (Fig. 6), likely due to the extremely low SNR at slab boundary slices. This issue poses a significant challenge for accurate slab profile estimation in NPEN, particularly in regions with strong B0 field inhomogeneity, such as lower slabs near the pons. A potential solution is to measure a slab profile using low-resolution high-SNR data and incorporate it as a prior in the NPEN correction. This strategy has shown efficacy in PEN (Van et al., 2015) using calibration scans with the same slab configuration and oversampling in the slice direction. In the original NPEN work (Wu, Koopmans, et al., 2016), a low-resolution *b =* 0 image from the central segment of a 3D rs-EPI scan with slice oversampling was used to initialize slab profile estimation, demonstrating robust performance. Furthermore, motion induced shifts during acquisition can exacerbate slab boundary artifacts by spreading them to more slices, as the anatomical region intersecting the slab edge changes over time. NPEN assumes a fixed slab excitation profile within a volume and does not account for such motion-induced profile drift. Consequently, intra-volume motion violates this assumption, reducing NPEN’s accuracy and effectiveness. In Fig. 6, as quantified from each subject’s “eddy” output, Subject 2 exhibited substantially more motion than Subject 1 (mean root-mean-square displacement: 1.56 mm vs. 0.72 mm; mean translation along the slice direction: 1.28 mm vs. 0.19 mm). This may have contributed to the more prominent slab boundary artifacts observed in Subject 2. In future scans, improved motion prevention strategies, such as enhanced padding and personalized head stabilizer (Wang et al., 2021) might help mitigate this issue.

## 5 Conclusion

In this work, an acquisition and reconstruction framework is proposed to achieve high-quality submillimeter dMRI 0.53–0.65 mm isotropic resolutions for in-vivo human brains. Comprehensive in-vivo experiments demonstrate the adequate SNR, high image sharpness, and excellent anatomical fidelity of the submillimeter data. Our data are able to resolve small subcortical structures, reduce the gyral bias, and improve U-fiber mapping compared to data at conventional resolutions (1.05–1.22 mm). Our method is robust at 7T, demonstrating excellent agreement with previous post-mortem data at 0.5 mm resolution acquired from the same scanner. Finally, by employing the scanner-agnostic, open-source implementation using Pulseq, we hope to improve the accessibility of our method to a broad range of scanning platforms and research laboratories.

## Supplementary Material

Supplementary material associated with this article can be found, in the online version, at doi: 10.1016/j.media.2025.103834.

Supplementary Material

## Figures and Tables

**Table 1:** Key parameters for submillimeter dMRI acquisition.

Res. (mm^3^)	B0	Matrix size	PF	TE1/TE2/TR (ms)^a^	*N_seg_/N_acq_* ^b^	ES_eff_ (ms)^c^	*T_acq_* per vol.	#b0/ DWI^d^	*T_acq_* in total	# sub^e^
0.65	3T	336x336x164	1	102/195/2500	6/6	0.21	6 min	2/6	48 min	1
0.53	3T	414x414x186	3/4	80/201/2000	6/3	0.22	2.7 min	4/20	65 min	2
0.61	7T	360x360x174	1	87/153/2600	8/8	0.15	7.6 min	2/6	61 min	1

a TE1 is the imaging echo time and TE2 is the navigator echo time.

b For the 3T 0.53 mm protocol, the 3 acquired ky segments are evenly spaced.

c ES_eff_ refers to the effective echo spacing (i.e., echo spacing/*N_seg_*).

d Diffusion-weighted images (DWI) are acquired at *b* = 1000 s/mm^2^. *b* = 0 data are acquired with opposite phase encoding directions to enable distortion correction.

e #sub: number of subjects scanned for the given protocol.

**Table 2:** Quantitative comparison of image reconstruction and processing strategies. The performance of SPIRiT, SPIRiT followed by standalone BM4D denoising (SPIRiT+denoising), and denoiser-regularized SPIRiT (DnSPIRiT) is evaluated using six metrics: SNR of the *b* = 0 image (SNR_b=0_), SNR of diffusion-weighted images (SNR_DWI_, averaged across six DWIs), angular contrast-to-noise ratio (CNR_DWI_), image sharpness (normalized Tenengrad, averaged across six DWIs), and the absolute bias in white matter fractional anisotropy (FA) and whole-brain mean diffusivity (MD) relative to the 1.22 mm reference. The best and second-best values for each metric are highlighted in **bold** and *italics*, respectively.

	SNR_b=0_	SNR_DWI_	CNR_DWI_	Sharpness	|ΔFA|	|ΔMD| (× 10^–5^ mm^2^/s)
SPIRiT	6.65	3.18	0.87	**0.50**	0.163	4.54
SPIRiT+denoising	**13.45**	**6.25**	**1.17**	0.28	*0.019*	*3.85*
DnSPIRiT	*11.19*	*5.29*	*1.04*	*0.39*	**0.002**	**3.62**

## Data Availability

The Pulseq sequence implementation and MATLAB reconstruction code will be made available upon reasonable request. We are currently unable to share subject level data because of data protection issues, although our center is actively working on a solution to this.
