# Supplementary material for: Submillimeter diffusion MRI using an in-plane segmented 3D multi-slab acquisition and denoiser-regularized reconstruction
Source: Med Image Anal. Author manuscript; Available in PMC 2026 Feb 18. (PMC7618738; doi:10.1016/j.media.2025.103834)
Supplement: Supplementary Material [file EMS212315-supplement-Supplementary_Material.pdf]

Submillimeter diffusion MRI using an in-plane segmented 3D multi-slab acquisition and denoiser-regularized reconstruction

Ziyu Li[1], Silei Zhu[1], Karla L. Miller[1*], Wenchuan Wu[1*]

[1]Oxford Centre for Integrative Neuroimaging, FMRIB, Nuffield Department of Clinical Neurosciences, University of Oxford, Oxford, United Kingdom.

* indicates equal contributions.

Correspondence to: Wenchuan Wu, Ph.D., FMRIB, John Radcliffe Hospital, Headington, Oxford, OX3 9DU, UK. E-mail: wenchuan.wu@ndcn.ox.ac.uk.

Supplementary Information

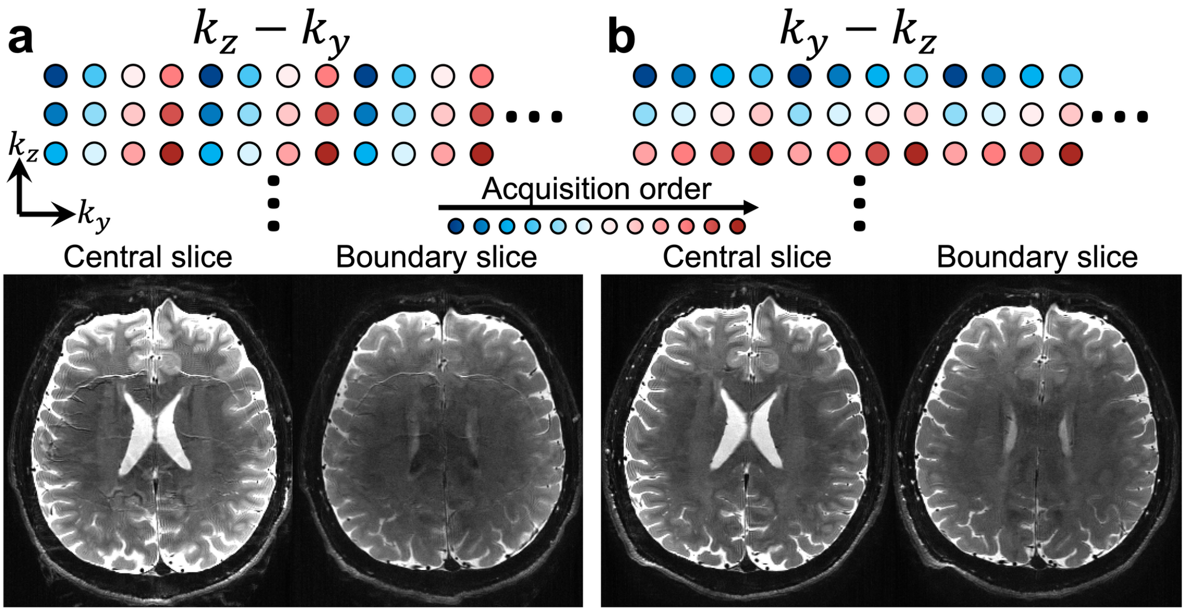

Supplementary Figure 1. Impact of the sampling order of in-plane segmented 3D EPI. The "kz-ky" (a, all kz planes for one ky segment are acquired before proceeding to the next segment) and "ky-kz" (b, all ky segments for one kz plane are acquired before proceeding to the next kz) sampling orders are illustrated with a simplified 4-segment example. Representative b=0 images from slab central and boundary slices (0.65 mm isotropic resolution, 6-segment acquisition) are demonstrated below the corresponding sampling orders.

Res. (mm^3)	#slab (slice)[a]	OS[b]	OL[c]	RF	Ext. / ref. pulse length	Ext. / ref. flip angle[d]	R_{nav}[e]
0.65	9 (20)	20%	2	SLR	6 / 9 ms	90° / 160°	4
0.53	8 (25)	8%	2	SLR	6 / 9 ms	90° / 160°	4
0.61	9 (20)	10%	2	sinc	6 / 8 ms	90° / 180°	6

Supplementary Table 1. Additional parameters for submillimeter dMRI acquisition. a. Number of slabs and number of slices within each slab (9 (20) indicates 9 slabs are acquired with 20 slices in each slab). b. Oversampling factor along kz to reduce boundary slice aliasing. c. Number of overlapped slices between adjacent slabs. d. Flip angle for the refocusing pulse is set to 160° for 0.65 mm and 0.53 mm protocols to reduce slab boundary saturation artifacts. e. The navigators are acquired for each shot with a lower parallel imaging acceleration factor for more robust reconstruction. Since the motion-induced phase is typically smooth, the distortion difference between the image and the navigator is not expected to significantly affect the reconstruction quality.

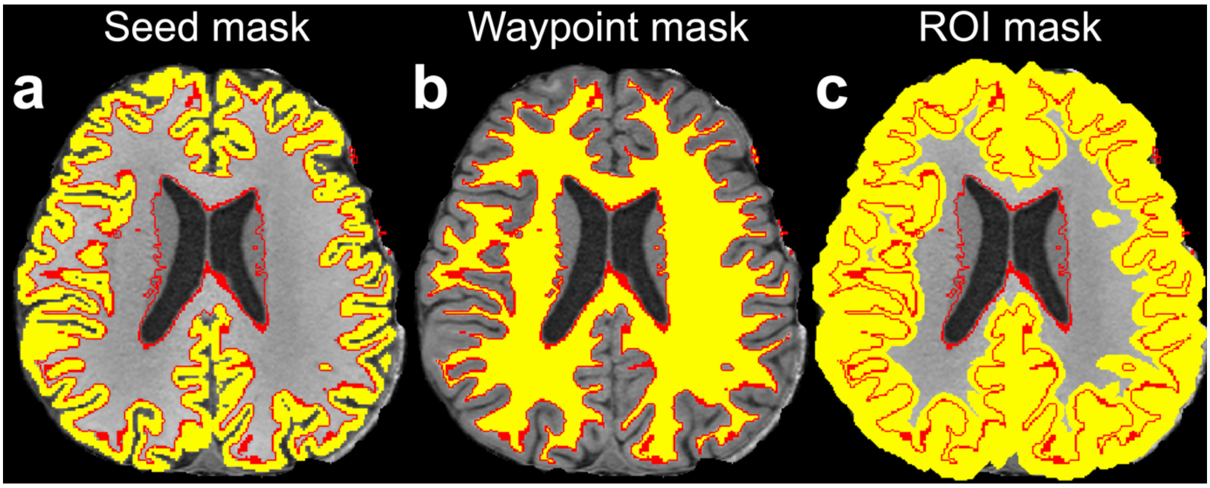

Supplementary Figure 2. Masks for tracking the short association fibers. The seed (a), waypoint (b), and region-of-interest (ROI) (c) masks (yellow) for tracking the short association fibers are displayed on an axial slice of the T1w image, with the white matter boundary marked in red and overlayed.

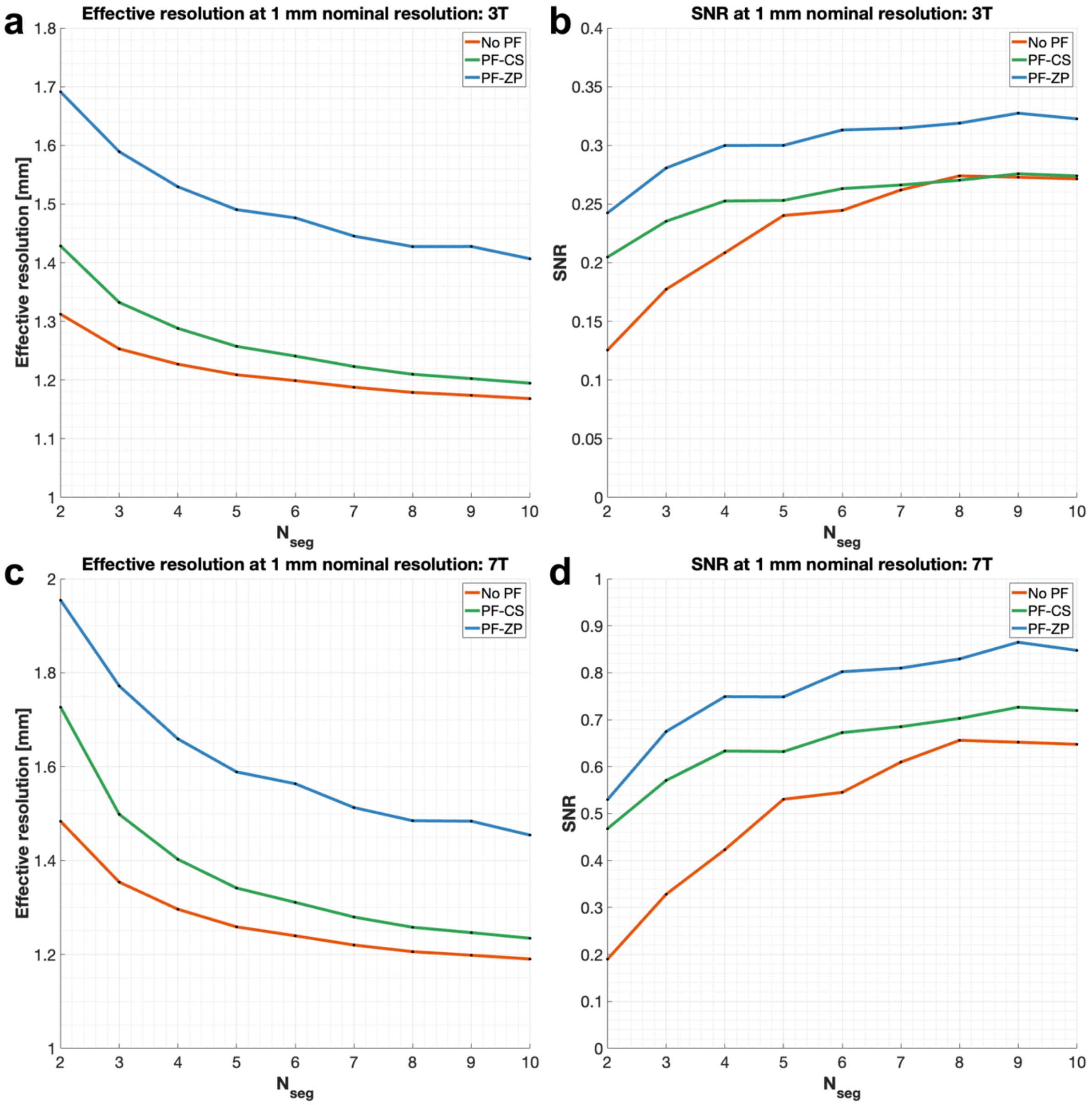

Supplementary Figure 3. Simulation of effective resolution and SNR for 1 mm diffusion-weighted EPI. Three sampling strategies are evaluated: no partial Fourier (No PF, red), 6/8 partial Fourier with conjugate symmetric filling (PF-CS, green), and 6/8 partial Fourier with zero padding (PF-ZP, blue). Simulations are conducted for 1 mm diffusion-weighted EPI at 3T (a, b) and 7T (c, d) for white matter, using TR=2.5 s, b-value=1000 s/mm^2, bandwidth=1384 Hz/pixel, and tissue relaxation parameters T1/T2/T2*=832/79.6/53.2 ms (3T) and 1220/47/26.8 ms (7T) for different in-plane segmentation numbers (N_{seg}).

	DWI 1 (1, 0, 0)	DWI 2 (0.45, 0.57, 0.69)	DWI 3 (-0.45, -0.83, 0.32)	DWI 4 (-0.45, 0.05, 0.89)	DWI 5 (-0.45, 0.86, 0.23)	DWI 6 (-0.45, 0.48, -0.75)
SPIRiT	3.18	3.20	3.13	3.20	3.20	3.18
SPIRiT+denoising	6.25	6.30	6.15	6.29	6.27	6.23
DnSPIRiT	5.29	5.33	5.21	5.32	5.31	5.28

Supplementary Table 2. SNR comparison across image reconstruction and processing strategies. The SNR of each individual diffusion-weighted image (DWI), along with its corresponding diffusion direction (listed below each volume), is reported for SPIRiT, SPIRiT followed by standalone BM4D denoising (SPIRiT+denoising), and denoiser-regularized SPIRiT (DnSPIRiT).

	DWI 1 (1, 0, 0)	DWI 2 (0.45, 0.57, 0.69)	DWI 3 (-0.45, -0.83, 0.32)	DWI 4 (-0.45, 0.05, 0.89)	DWI 5 (-0.45, 0.86, 0.23)	DWI 6 (-0.45, 0.48, -0.75)
SPIRiT	0.500	0.500	0.499	0.505	0.503	0.505
SPIRiT+denoising	0.285	0.277	0.282	0.277	0.284	0.283
DnSPIRiT	0.396	0.391	0.394	0.391	0.397	0.395

Supplementary Table 3. Image sharpness comparison across image reconstruction and processing strategies. The image sharpness (normalized Tenengrad) of each individual diffusion-weighted image (DWI), along with its corresponding diffusion direction (listed below each volume), is reported for SPIRiT, SPIRiT followed by standalone BM4D denoising (SPIRiT+denoising), and denoiser-regularized SPIRiT (DnSPIRiT).

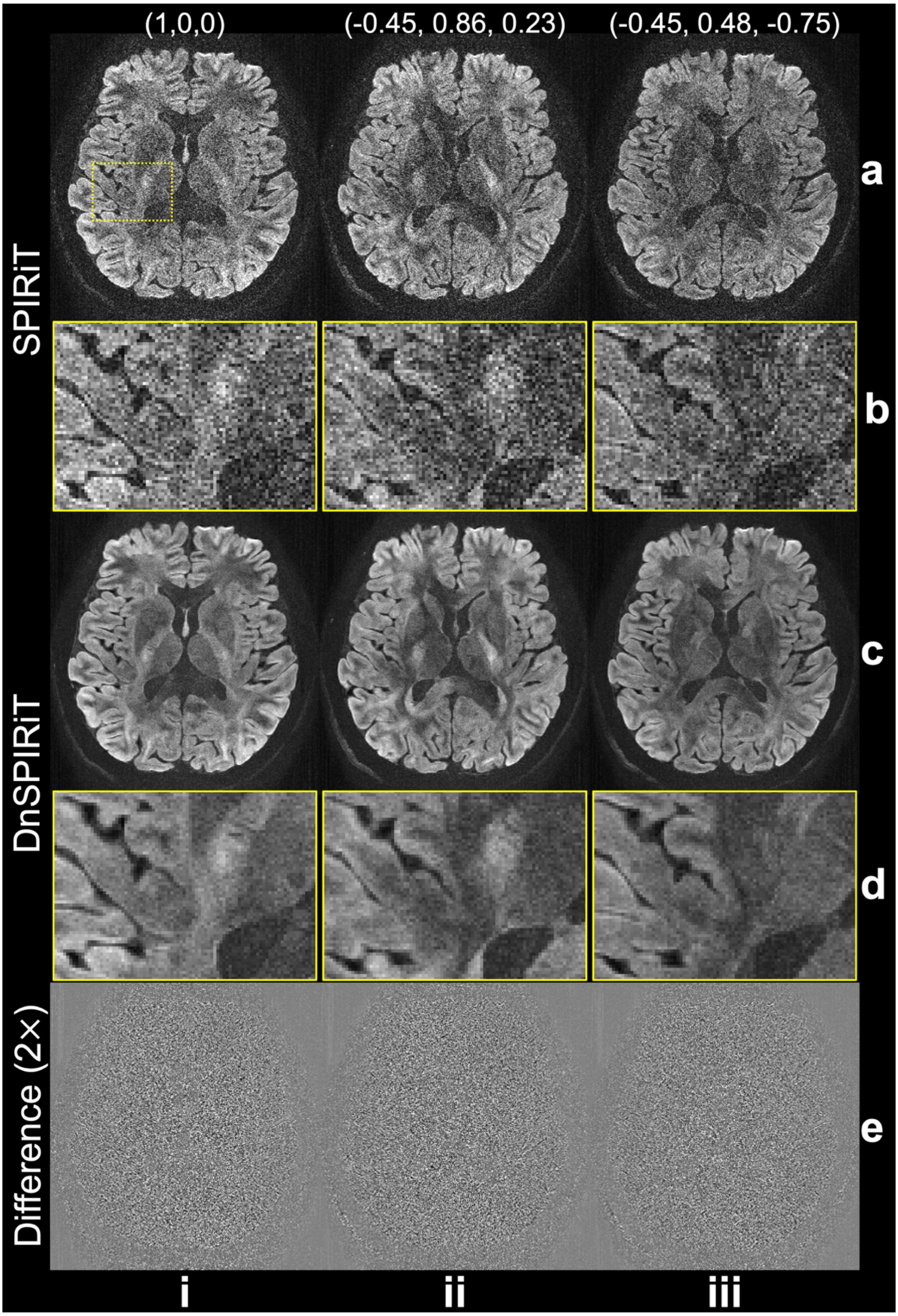

Supplementary Figure 4. Image reconstruction results of more directions. In-vivo diffusion-weighted data (b=1000 s/mm^2) from 3T 0.65 mm protocol along 3 representative diffusion encoding directions (i-iii, the direction is displayed above each image) are reconstructed using SPIRiT (a) and denoiser-regularized SPIRiT (DnSPIRiT, c), with an enlarged region showing the image detail (b, d). Their difference is also shown to demonstrate the removed noise (e).

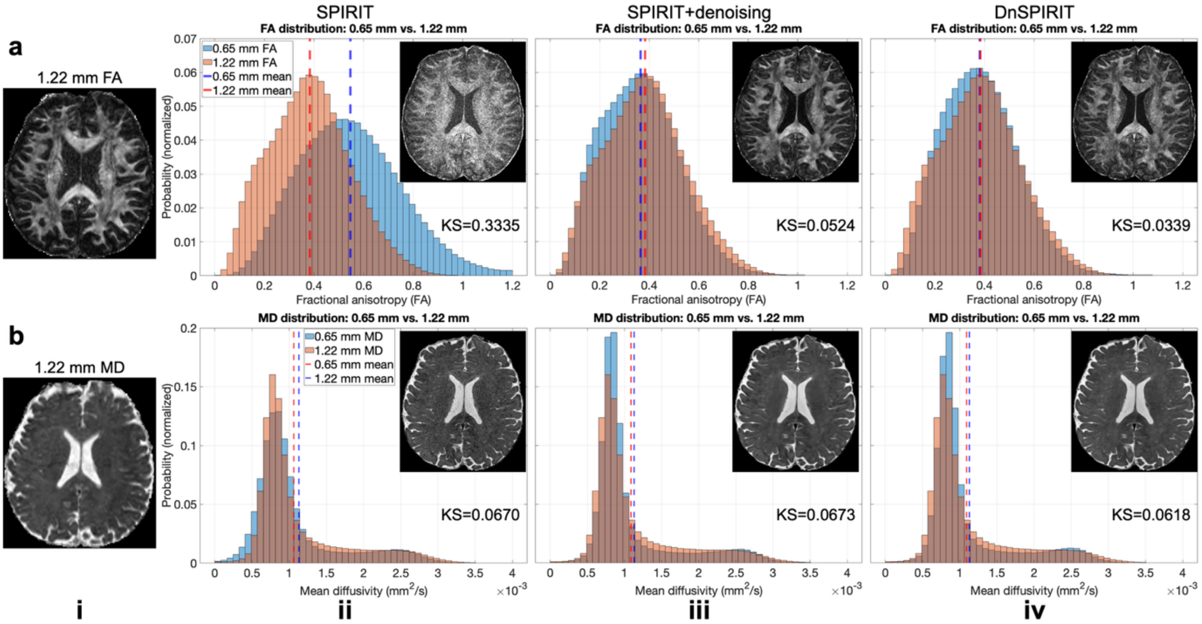

Supplementary Figure 5. Distribution comparisons of FA and MD across reconstruction and processing strategies. Fractional anisotropy (FA, a) and mean diffusivity (MD, b) maps from the 3T 0.65 mm protocol reconstructed using SPIRiT (ii), SPIRiT followed by standalone BM4D denoising (SPIRiT+denoising, iii), and denoiser-regularized SPIRiT (DnSPIRiT, iv) are compared against the 1.22 mm reference (i). Distributions of FA (computed within a T1w-derived white matter mask) and MD (computed within a whole-brain mask) are shown as histograms of voxel-wise values alongside the images. Similarity to the reference 1.22 mm data distribution is quantified using the Kolmogorov-Smirnov (KS) statistic, with lower values indicating closer agreement.

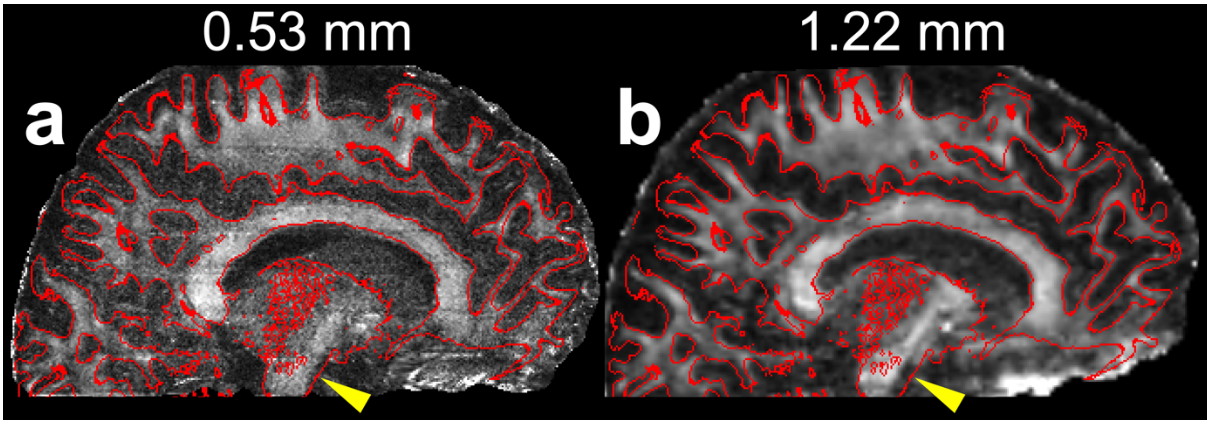

Supplementary Figure 6. Anatomical fidelity comparison between 0.53 mm and 1.22 mm data. Fractional anisotropy maps with gray-white matter boundary derived from the T1w image using FSL's "fast" overlayed for 0.53 mm (a) and 1.22 mm (b) data. The 0.53 mm data show better anatomical fidelity due to shorter effective echo spacing, especially in regions with strong field inhomogeneity (e.g., pons, yellow arrows).

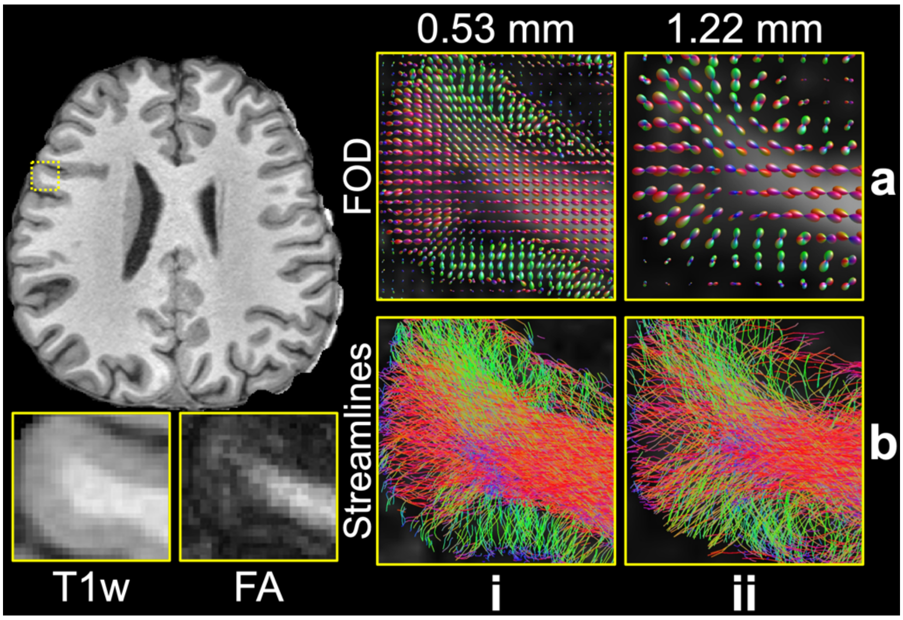

Supplementary Figure 7. Fiber distributions at a large gyrus. The fiber orientation distributions (FOD) (a) and tractography streamlines (b) for a large gyrus from the 0.53 mm (i) and 1.22 mm (ii) data are shown. At this large gyrus, the gyral bias problem is less pronounced even on 1.22 mm data. Co-registered T1w images, along with enlarged T1w and fractional anisotropy (FA) maps (0.53 mm isotropic resolution) for the selected gyri, are provided as anatomical references to aid visualization of the cortical structure.

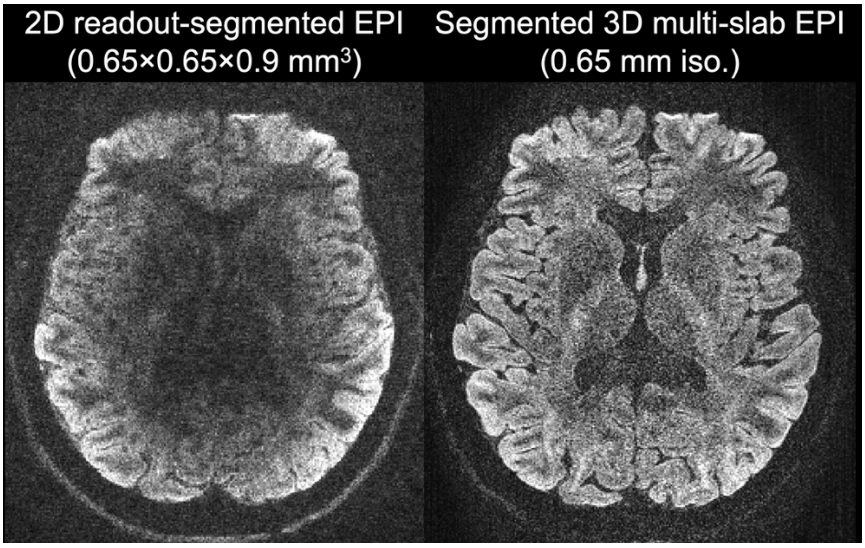

Supplementary Figure 8. Comparison between 2D and 3D acquisitions. Images acquired with Siemens product 2D readout-segmented EPI (rs-EPI, scanner reconstruction) and our proposed segmented 3D multi-slab EPI (SPIRiT reconstruction) with a matched diffusion encoding direction (1, 0, 0) and b=1000 s/mm^2 are presented. The 2D rs-EPI acquisition uses the following parameters: 5 segments, TE/TR = 91/25700 ms, GRAPPA = 3, echo spacing = 0.56 ms, resolution = 0.65×0.65×0.9 mm^3, total scan time per volume = 128.5 s, averaged over 3 repetitions (total acquisition ~6.4 min). The segmented 3D multi-slab EPI acquires a single volume with 0.65 mm isotropic resolution in 6 minutes, resulting in substantially higher SNR compared to the 2D acquisition.

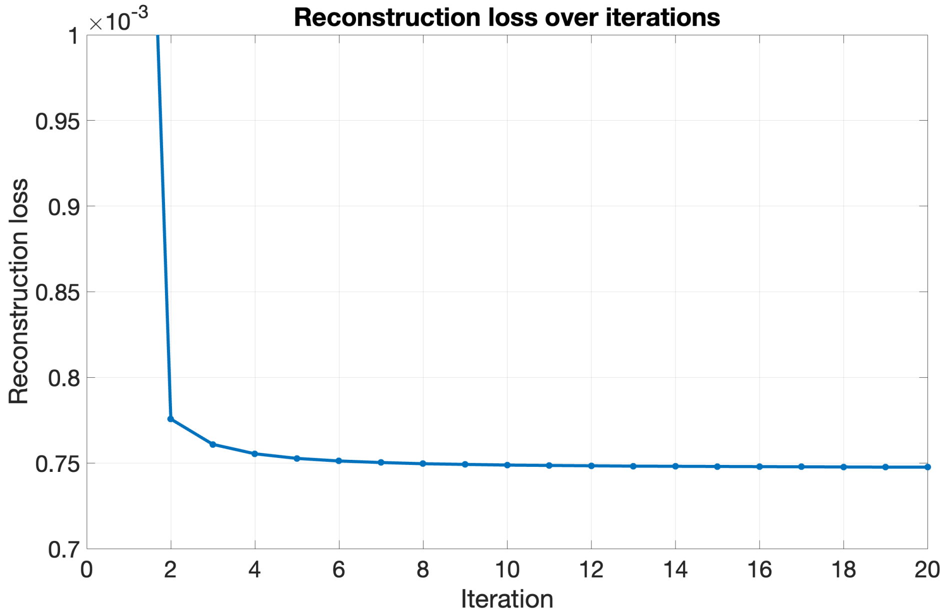

Supplementary Figure 9. Reconstruction loss over iterations for DnSPIRiT. The plot shows the reconstruction loss of DnSPIRiT using BM4D as the denoiser over 20 iterations, applied to 3T 0.65 mm data. The loss rapidly decreases in the initial iterations and gradually stabilizes, indicating good empirical convergence of the reconstruction.

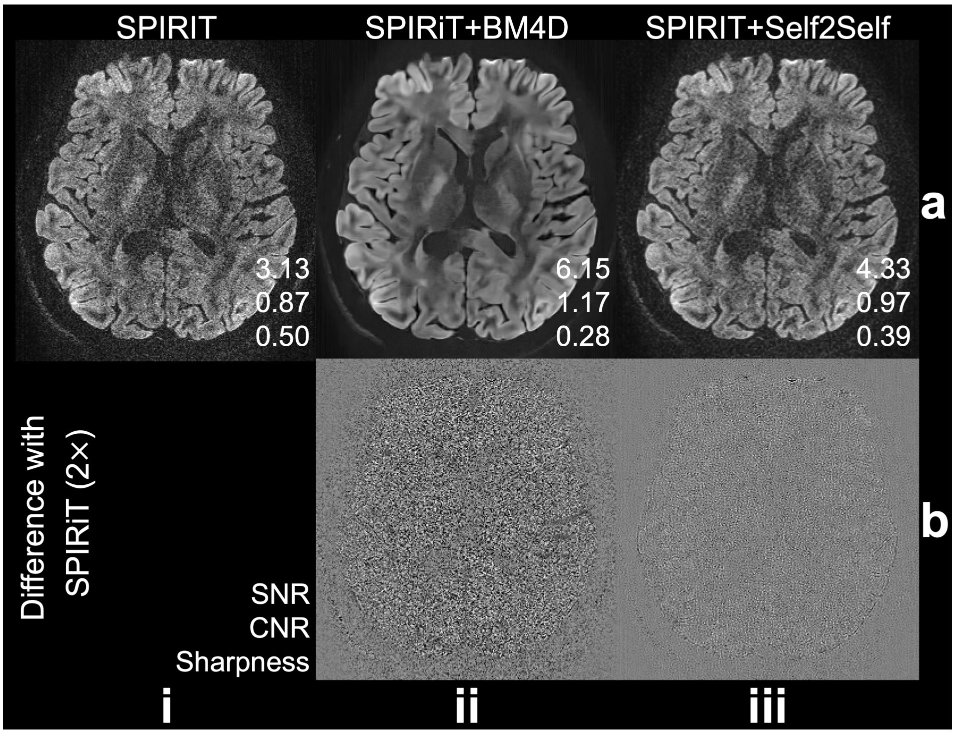

Supplementary Figure 10. Evaluation of deep learning denoising on submillimeter dMRI. In-vivo diffusion-weighted images (DWIs) (b = 1000 s/mm^2) from the 3T 0.65 mm protocol along the direction (–0.45, –0.83, 0.32) were reconstructed using (i) SPIRiT, (ii) SPIRiT followed by BM4D denoising, and (iii) SPIRiT followed by Self2Self denoising. Self2Self was trained on 2D slices from six DWIs and two b = 0 volumes for 100 epochs using a learning rate of 2e-5. The network architecture follows the original implementation, with a higher masking rate (m = 0.5) and dropout rate (d = 0.5) to better handle high noise levels. The difference maps between SPIRiT and denoised images are also shown to visualize the noise and structural content removed by each denoiser (b). SNR, angular contrast-to-noise ratio (CNR), and image sharpness (normalized Tenengrad) are listed for each image to quantify the image quality.
